# Dataset Fragmentation, Cognitive Variability, and Reproducibility Challenges in EEG-Based Brain–Computer Interfaces: A PRISMA-Based Systematic Review

**DOI:** 10.3390/s26175562

**Published:** 2026-09-01

**Authors:** Janis Peksa

**Affiliations:** 1 Information Technology Faculty, Turiba University, Graudu Street 68, LV-1058 Riga, Latvia; janis.peksa@turiba.lv or janis.peksa@rtu.lv; 2 Institute of Information Technology, Riga Technical University, Kalku Street 1, LV-1658 Riga, Latvia

**Keywords:** electroencephalography, brain–computer interfaces, dataset interoperability, semantic metadata, reproducibility, cognitive variability, cross-dataset learning, transfer learning

## Abstract

**Highlights:**

**What are the main findings?**

- EEG-BCI dataset fragmentation spans structural, semantic, procedural, computational, and human-contextual dimensions; common file access or statistical alignment alone does not establish dataset comparability.
- Within the curated 129-publication synthesis corpus, the median unweighted reporting-transparency score was 9/10, while code or pipeline availability was stated in only 20.9% and cognitive and user-state metadata were represented inconsistently.

**What are the implications of the main findings?**

- Scalable cross-dataset learning requires analysis-dependent compatibility rules, explicit provenance, contextual metadata and auditable transformations, rather than relying on format conversion alone.
- Existing standards, software platforms, benchmark frameworks, ontologies and transfer-learning methods should be connected as complementary interoperability layers, with uncertainty and information loss reported explicitly.

**Abstract:**

Public EEG-based brain–computer interface (BCI) datasets are expanding rapidly, yet differences in sensors, experimental protocols, task/event semantics, preprocessing, participant context, and evaluation limit reproducibility and cross-dataset learning. This review examined whether heterogeneous EEG-BCI resources can support reproducible analysis across sources. A PRISMA-based systematic mapping review of literature published from 2014 to June 2026 was conducted across Scopus, Web of Science Core Collection, IEEE Xplore, PubMed, and ACM Digital Library, yielding 16,920 records. Following screening and evidence-focused curation, a curated synthesis corpus of 129 publications was retained and confirmed by full-text review. Evidence was coded across structural, semantic, procedural, human/contextual, and computational fragmentation, and reporting transparency was assessed using ten criteria. The synthesis identified heterogeneity in acquisition, channel layouts, task/event definitions, preprocessing, participant/session context, and evaluation design. Existing standards, ontologies, software platforms, benchmark frameworks, and transfer-learning methods address complementary layers but do not provide complete semantic interoperability. Within the retained corpus, the median transparency score was 9/10; data availability was stated in 61.2% and code or pipeline availability in 20.9%. These frequencies describe the curated corpus rather than the field as a whole. Scalable cross-dataset analysis requires analysis-dependent compatibility rules, explicit provenance, contextual metadata, and auditable transformations that preserve dataset identity, uncertainty, and information loss.

## 1. Introduction

Electroencephalography-based brain–computer interfaces (EEG-BCIs) translate neural activity patterns into commands or user-state estimates, bypassing conventional neuromuscular pathways. While the field spans well-established paradigms, such as motor imagery, event-related potentials, and passive BCIs, recent years have seen a surge in publicly accessible datasets [1,2,3,4,5,6,7,8,9,10,11,12,13]. Although this growth encourages systematic benchmarking and cross-participant learning, it has also exposed inconsistencies in how EEG-BCI data are acquired, processed, and evaluated.

These inconsistencies are particularly consequential for data-intensive machine learning. While deep neural networks and generalized transfer-learning models could be trained on large, diverse training corpora, most BCI studies remain highly isolated by specific laboratories, participant cohorts, or paradigms [14,15,16,17]. Unified benchmarking frameworks such as the Mother of All BCI Benchmarks (MOABB) and cross-subject transfer studies have demonstrated that data reuse is possible under the right conditions [14,18,19,20,21,22,23,24,25]. However, simply having access to multiple datasets does not guarantee they can be merged without substantial interpretation and transformation.

EEG-BCI datasets diverge across several interconnected layers. Structural discrepancies manifest in file formats and sensor layouts, while experimental variations affect factors ranging from stimulus timing to participant instructions. Routine processing pipelines introduce significant procedural variance. At the semantic level, identical paradigm labels frequently mask profoundly different experimental protocols and user intentions [26,27,28,29,30]. As a result, forcing signals into a shared file structure does not inherently establish that the datasets carry equivalent meaning.

This multidimensional fragmentation directly undermines reproducibility. Because EEG outcomes are highly sensitive to seemingly minor preprocessing choices [31,32,33], true reproduction is often blocked by incomplete documentation of software versions, model parameters, and cross-validation strategies [15,34,35,36,37]. Therefore, public data availability is only a baseline requirement; a downloadable dataset remains essentially unusable if its experimental context, processing lineage, and evaluation assumptions are not transparently defined.

The heterogeneity of EEG-BCI data is not exclusively technical or procedural. Neural signals and BCI performance vary substantially between participants and across recording sessions [38,39,40]. Mental fatigue can alter spectral and event-related responses during prolonged BCI use [41,42,43,44,45,46,47,48,49,50,51]. Workload, vigilance, and stress have been studied in passive and adaptive BCI settings, including cross-task, cross-session, and realistic operational conditions [52,53,54,55,56,57,58,59,60,61,62,63,64]. Motivation, mental state, demographic and functional differences, and broader inter-subject factors may likewise affect task engagement and decoding performance [38,39,40,65,66,67,68]. These factors are often treated as noise or unexplained participant variability. From a dataset-interoperability perspective, however, they form part of the context in which the signals were generated. Two datasets collected with similar hardware and nominally identical tasks may still differ systematically because participants were exposed to different experimental conditions, including training procedures, session durations, cognitive demands, feedback conditions and levels of fatigue. When such information is missing or represented inconsistently, it becomes difficult to determine the source of cross-dataset performance differences: neural variability, acquisition conditions, experimental design, or computational methodology.

Several initiatives address important parts of this problem. Containerized processing environments can support repeatable large-scale EEG analysis [69]. EEG-BIDS provides a structured approach to organizing EEG data and associated metadata [26]. Ontological and common-model approaches seek to describe BCI systems, data elements and contextual relationships more consistently [27,29,70]. Open-source platforms and reusable software frameworks support important components of EEG-BCI systems, including common acquisition, processing, deployment, and evaluation workflows [71,72]. MOABB and related benchmark libraries improve access to multiple datasets and encourage the comparison of algorithms through shared pipelines [14,18,19]. These developments represent essential foundations for reproducible BCI research, but they operate at different levels and should not be treated as interchangeable. A data-organization specification, an ontology, an experimental platform, and a benchmark interface address different aspects of interoperability. Important limitations consequently remain. Datasets with different numbers of channels or sensor configurations may require explicit dimensional alignment or interpolation before they can be merged [28,73]. P300 datasets may use incompatible event definitions, stimulus structures, or class-label conventions even when they address the same broad spelling paradigm [30]. Normalization and alignment choices influence the success of domain adaptation [32], while deep-learning performance may deteriorate substantially when models are transferred between independently collected datasets [74]. Thus, technical readability, common software access, statistical alignment, and semantic compatibility are distinct properties in EEG-BCI systems. A framework may make several datasets loadable through one interface without establishing whether their tasks, events, participant states, acquisition contexts, and processing histories are sufficiently comparable for a given scientific analysis. Common access should therefore not be interpreted as evidence of scientific comparability.

Recognizing this distinction is crucial for scalable learning. While an extensive body of research has explored cross-subject transfer, domain adaptation, data alignment and calibration reduction [21,22,23,24,25,75,76,77,78,79,80,81,82,83,84,85,86,87,88,89,90,91,92,93,94,95,96,97,98,99,100,101,102,103,104,105,106,107,108], more recent work has explicitly expanded into cross-dataset transfer and source selection [109,110,111,112,113]. These methods primarily aim to mitigate distributional differences and minimize the need for participant-specific calibration. However, statistical adaptation alone cannot resolve deeper ambiguities in task meaning, event definitions, sensor configurations, or preprocessing provenance. A model might successfully minimize a measurable distribution shift while still relying on datasets with only partially aligned experimental semantics. At the same time, two thoroughly documented datasets might be semantically compatible, yet remain unsuitable for direct pooling if differences in sensor layouts, signal quality, or participant populations introduce irreducible barriers. Ultimately, the core scientific challenge is not achieving universal format conversion, but rather determining which dataset components are naturally compatible, which demand explicit transformation, and which establish strict boundaries for interoperability.

Previous reviews have examined individual parts of this landscape. Transfer learning and domain adaptation in EEG-BCI have been reviewed from methodological and tutorial perspectives [22,24,25], while separate reviews have considered calibration reduction and short- or zero-calibration operation [79,80]. Inter- and intra-subject variability has been synthesized for sensorimotor BCIs [38], and recent reviews have addressed fatigue or broader participant factors affecting BCI use [48,68]. These contributions provide important domain-specific evidence, but they are generally organized around a method, paradigm, or user factor rather than around the compatibility of heterogeneous datasets as scientific objects. Consequently, they do not jointly examine how dataset organization, experimental semantics, processing provenance, cognitive and user context, reproducibility, and learning-oriented generalization constrain one another.

The contribution of the present review is therefore fourfold. First, it treats EEG-BCI dataset fragmentation as a multidimensional problem spanning structural, semantic, procedural, human/contextual, and computational layers rather than as a file-format problem alone. Second, it treats cognitive and user variability as part of the context that gives a dataset its analytical meaning, not merely as nuisance variance. Third, it distinguishes technical accessibility, metadata completeness, semantic compatibility, reproducibility, statistical alignment, and practical learnability as related but non-equivalent properties. Fourth, it connects these layers to scalable cross-dataset learning by framing compatibility as analysis-dependent: datasets may be directly comparable for one purpose, require explicit transformation for another, or remain unsuitable when essential information is absent. This integrated perspective addresses a dimension not jointly covered by reviews centered on transfer algorithms, calibration reduction, individual variability factors, or classification performance [22,24,25,38,48,68,79,80].

Against this background, a systematic synthesis across these areas is needed to determine which forms of heterogeneity can be harmonized, which require explicit and auditable transformation, and which define boundaries for reuse. Fragmentation must therefore be characterized beyond file formats to include task semantics, experimental protocols, processing provenance, evaluation design, and human context. Existing tools and standards must likewise be evaluated according to the interoperability layer they address, while cross-subject and cross-dataset learning should be interpreted in relation to the heterogeneity those methods attempt to overcome. Without this integrated perspective, generalization failures risk being attributed primarily to algorithm design even when incompatibilities in data generation, representation, or meaning are decisive.

Accordingly, this study presents a PRISMA-based systematic mapping review of EEG-BCI literature published between 2014 and 2026. This systematic mapping review was conducted and reported in accordance with the PRISMA 2020 statement [114] and addresses five connected questions concerning dataset fragmentation, reproducibility, cognitive and user variability, current interoperability solutions, and scalable learning. The review protocol was not prospectively registered. A mapping design was selected because the evidence encompasses heterogeneous dataset papers, benchmarking studies, reproducibility analyses, cognitive-variability research, standards and software frameworks, and transfer-learning methods rather than a common intervention or outcome suitable for meta-analysis.

Within this scope, the review maps EEG-BCI fragmentation across structural, semantic, procedural, human/contextual, and computational dimensions; examines how incomplete metadata, processing ambiguity, and evaluation choices constrain reproducibility; and distinguishes the roles and limitations of current standards, software platforms, benchmark frameworks, harmonization methods, and transfer-learning approaches. The synthesis develops the argument that scalable cross-dataset learning requires layered, context-aware interoperability rather than format conversion alone. Its purpose is not to propose another universal data format or to assume that all heterogeneous datasets can be made equivalent, but to identify the information, provenance, and compatibility conditions that must be explicit before cross-dataset reuse is scientifically defensible.

## 2. Materials and Methods

### 2.1. Review Design and Scope

The study was designed as a PRISMA-based systematic mapping review. A mapping approach was selected because the literature covered several connected but methodologically distinct areas: EEG dataset organization, metadata and data standards, reproducibility and benchmarking, cognitive and user variability, and cross-dataset learning. The purpose was therefore not to estimate a pooled intervention effect, but to identify the principal forms of fragmentation in EEG-based brain–computer interface research, classify the solutions proposed in the literature, and determine where important interoperability gaps remain.

The review was organized around the following scientific sequence:

dataset fragmentation → reproducibility → cognitive and user variability → semantic interoperability → scalable learning.

This sequence reflects the observation that technical differences between EEG datasets cannot be considered separately from experimental design, preprocessing, user state, and evaluation procedures. Existing initiatives address parts of this problem through standardized EEG organization, ontological descriptions, shared BCI models, reusable software, common benchmark pipelines, and reproducible reporting practices [14,15,16,18,19,26,27,29,31,36,69,70,71,72]. However, the literature also shows that converting data into a common storage structure does not necessarily make datasets semantically comparable. Differences in event meaning, task instructions, channel configurations, preprocessing history, subject characteristics, and recording context may persist after format-level harmonization [28,30,32,33,35,37,73,115].

The unit of analysis in this review was an individual scholarly publication. Journal articles and peer-reviewed conference papers were eligible. Reviews were retained when they offered a direct synthesis of one of the review questions, whereas software, standards, and dataset papers were included when they contributed evidence about data organization, metadata, reproducibility, interoperability, or cross-dataset reuse.

The review period extended from 1 January 2014 to 27 June 2026. The starting year was chosen to capture the recent expansion of public EEG datasets, open benchmarking environments, transfer-learning methods, passive BCI research and modern data-sharing practices. Older foundational BCI systems were considered only as contextual background and were not part of the principal screening corpus.

After completion of the searches, screening, evidence-focused curation, full-text confirmation, data extraction, coding, transparency assessment, and synthesis, the implemented review protocol and methodological record were retrospectively registered with the Open Science Framework (OSF) (https://doi.org/10.17605/OSF.IO/VS5FW). The registration records the methods actually used and the deviations from the initially intended screening workflow and should not be interpreted as prospective preregistration.

### 2.2. Review Questions

Five review questions guided all processes in this research: the search, screening, extraction and synthesis.

RQ1: Dataset fragmentation. How is fragmentation characterized across the technical, structural, experimental, and semantic dimensions of EEG-based BCI datasets?

RQ2: Reproducibility. Which dataset, preprocessing, reporting, and evaluation practices limit the reproducibility and comparability of EEG-based BCI studies?

RQ3: Cognitive and user variability. How are temporal and inter-subject variations represented, and how do studies evaluate shifting user states such as cognitive workload, fatigue, attention, and motivation?

RQ4: Interoperability solutions. To what extent do current data standards, ontological models, repositories, and software frameworks address BCI interoperability, and what core limitations remain unresolved?

RQ5: Scalable learning. How does dataset heterogeneity affect scalable learning across subjects, sessions, and independently collected datasets, particularly through transfer learning, domain adaptation, source selection, and calibration-reduction approaches?

The scope of RQ1 and RQ4 was informed by work on containerized EEG analysis, MOABB, EEG-BIDS, BCI ontologies, IEEE BCI data models, dataset merging, and common system descriptions [18,26,27,28,29,30,69,70,71]. Reproducibility was interpreted broadly enough to include preprocessing sensitivity, reporting completeness, evaluation design, cross-validation choices, software availability, and the comparability of benchmark results [14,15,18,19,31,33,34,35,36,37]. Cognitive variability was treated as a component of dataset meaning rather than as an independent usability topic, because fatigue, workload, motivation, vigilance, and inter-subject differences can alter both neural measurements and BCI performance [38,39,40,41,44,47,48,49,51,64,65,66,68]. Scalable learning included studies that explicitly transferred or generalized information across participants, sessions, independently collected datasets, acquisition environments, or health states, including cross-dataset source-selection and calibration-reduction settings [21,22,24,25,74,79,80,103,108,109,110,111,112,113,116].

### 2.3. Eligibility Criteria

A publication was eligible for the principal corpus when it satisfied all general criteria and at least one thematic criterion.

The general inclusion criteria were:

1. the study involved non-invasive EEG or an EEG-containing multimodal BCI;
2. the study concerned human BCI research, BCI datasets, BCI benchmarking, or methods intended for BCI generalization;
3. the publication date fell within the defined search period;
4. the article was written in English;
5. the record represented a peer-reviewed journal article or conference paper;
6. sufficient bibliographic information was available to determine eligibility and support the planned extraction fields.

At least one of the following thematic criteria also had to be met:

- presentation, comparison, integration, or critical analysis of one or more EEG-based BCI datasets;
- explicit analysis of metadata, data formats, channel systems, event annotations, preprocessing lineage, ontologies, data models, or data-sharing practices;
- investigation of reproducibility, benchmarking, cross-validation, preprocessing sensitivity, open science, code availability, or standardized evaluation;
- analysis of inter-subject, inter-session, cognitive, motivational, attentional, workload, fatigue, vigilance, or related user-state variability;
- cross-subject, cross-session, cross-device, cross-center, or cross-dataset learning;
- transfer learning, domain adaptation, calibration reduction, dataset alignment, or generalization across heterogeneous BCI conditions.

Dataset papers were not included solely because they made EEG data publicly available. They also had to contribute to at least one review question through: benchmark value, multi-session or multi-subject structure, user-state information, acquisition heterogeneity, reusable metadata, explicit support for comparative analysis. Representative eligible dataset contributions included benchmark datasets for SSVEP, motor imagery, RSVP and P300, passive BCI, wearable acquisition, multi-day recording, and real-world operation [1,2,3,4,5,6,7,8,9,10,11,12,13,117,118,119,120,121,122,123,124,125,126,127,128,129,130].

The principal exclusion criteria were:

- invasive-only BCI;
- clinical EEG diagnosis without an operational or methodological relationship to BCI;
- sleep, epilepsy, emotion, or general cognitive EEG studies without a clear BCI context;
- non-human research;
- hardware-only studies without implications for dataset structure, reproducibility, user variability, or interoperability;
- individual classifier papers evaluated on a single dataset when they offered no evidence concerning transfer, reproducibility, benchmarking, calibration, or dataset reuse;
- papers in which terms such as attention or metadata referred only to a neural-network mechanism or an application-specific annotation unrelated to the review questions;
- non-English publications;
- editorials, abstracts, unpublished manuscripts, and other non-peer-reviewed items used only as background; and
- duplicate or superseded records.

Clinical and rehabilitation studies were not excluded automatically. They were retained when they provided evidence about variability across health states, calibration, transfer, dataset structure, or ecological validity. The same principle was applied to wearable and passive BCI studies: the decisive criterion was their contribution to the review questions rather than the application domain itself.

### 2.4. Information Sources and Search Strategy

Searches were conducted in Scopus, Web of Science Core Collection, IEEE Xplore, PubMed, and the ACM Digital Library. Four search families were developed to cover the five review questions without relying on a single excessively restrictive Boolean expression.

To capture the specific dimensions of dataset fragmentation and interoperability (Search Family A), the baseline query was:

(“brain-computer interface” OR “brain computer interface” OR BCI)

AND (EEG OR electroencephalograph*)

AND (dataset* OR “data set*” OR database* OR repository OR benchmark*)

AND (fragmentation OR interoperab* OR standard* OR metadata OR “data format” OR “file format” OR harmonization OR harmonisation).

For reproducibility and benchmarking literature (Search Family B), the query was structured as follows:

(“brain-computer interface” OR “brain computer interface” OR BCI)

AND (EEG OR electroencephalograph*)

AND (reproducib* OR benchmark* OR “open science” OR “open data” OR FAIR OR replicab*)

AND (dataset* OR pipeline* OR preprocessing OR evaluation).

To identify cross-dataset and cross-subject learning studies (Search Family C), the search was adapted to:

(“brain-computer interface” OR “brain computer interface” OR BCI)

AND (EEG OR electroencephalograph*)

AND (“cross-dataset” OR “cross dataset” OR “cross-subject” OR “cross subject” OR “transfer learning” OR “domain adaptation” OR generalization OR generalisation OR calibration).

Finally, the query for cognitive and user variability (Search Family D) took the following form:

(“brain-computer interface” OR “brain computer interface” OR BCI)

AND (EEG OR electroencephalograph*)

AND (“cognitive load” OR workload OR fatigue OR attention OR motivation OR “mental state” OR “user state” OR “inter-subject variability” OR “session-to-session variability”).

The syntax was adapted to the indexing structure of each database. Scopus searches were applied to titles, abstracts, and keywords. Web of Science searches used the Topic field. PubMed searches were restricted primarily to title and abstract terms and included a publication-date filter. IEEE Xplore and ACM Digital Library required shorter database-specific expressions because their interfaces handled long Boolean strings differently. The exact database queries, search dates, result counts, and export filenames were recorded in the search log and retained as part of the review audit trail.

Searches began on 12 February 2026, with additional runs, refinements, and updates conducted through June 2026. Search Family A was last run in Scopus on 12 June 2026. The remaining Scopus searches, the IEEE Xplore searches, and the PubMed searches were completed on 23 June 2026. Web of Science and ACM searches were completed on 27 June 2026.

No general web search was used as a substitute for the five database searches. Publisher pages, DOI records, PubMed pages, and institutional bibliographic records were consulted only to verify publication details, resolve ambiguous records, or check potentially important studies discovered during screening.

### 2.5. Record Management and Deduplication

All search outputs were imported into a structured screening workbook. Each occurrence retained its database source, search family, export filename, DOI, title, authors, year, publication source, abstract where available, keywords, and source URL. This provenance information was preserved even when multiple occurrences were merged into a single bibliographic record.

Deduplication was carried out in several stages. Deduplication began by normalizing DOI strings: URL prefixes were removed, strings were converted to lowercase, and trailing punctuation was deleted, allowing records sharing an identical DOI to be merged reliably. For records lacking a valid DOI, normalized titles were used for comparison. Title normalization included lowercasing, Unicode normalization, removal of punctuation, replacement of typographic dashes, and consolidation of repeated whitespace. Author, year, journal, and conference information were then used to review uncertain title matches.

When duplicate records contained different levels of detail, the most complete bibliographic version was retained as the primary record. Database provenance and search-family membership from the secondary records were appended to that record. Near-duplicates were inspected manually when titles differed only by capitalization, punctuation, abbreviated conference wording, or minor subtitle variation. A final duplicate check was repeated during the focused second-stage screening, because records that appeared distinct in large exports could still represent the same publication.

This procedure was deliberately conservative. Records were merged only when DOI or bibliographic evidence indicated that they referred to the same publication. Different articles using the same dataset, conference and journal versions with substantively different content, and later methodological extensions were not treated as duplicates unless the available evidence showed that one superseded the other.

### 2.6. Screening Procedure

Record selection proceeded through three connected stages: an initial screening of titles, abstracts and metadata; an evidence-focused secondary selection; and a final full-text verification of the synthesis corpus. These stages are reported separately to clearly distinguish the secondary relevance-based curation from a standard PRISMA full-text eligibility assessment.

#### 2.6.1. Initial Title, Abstract, and Metadata Screening

The first stage emphasized sensitivity. Titles, abstracts, author keywords, index key-words, document type, and publication source were assessed against the eligibility criteria. When an abstract was unavailable, the decision was based on the title and available citation metadata. Each record was classified as Include, Unclear, Background, or Exclude.

Each record received one of four preliminary decisions:

- Include: direct relevance to one or more review questions;
- Unclear: insufficient information for a confident decision;
- Background: useful contextual source that was not part of the principal evidence corpus;
- Exclude: failure to satisfy the eligibility criteria.

Exclusion decisions were coded using predefined reasons:

- E1: not an EEG-based BCI study;
- E2: not related to datasets, interoperability, reproducibility, variability, or scalable learning;
- E3: single-dataset algorithm paper without sufficient broader relevance;
- E4: invasive-only BCI;
- E5: full text or sufficient information unavailable;
- E6: insufficient methodological detail;
- E7: duplicate or superseded study;
- E8: non-English publication;
- E9: non-peer-reviewed or background-only material.

The single-dataset exclusion (E3) was applied judiciously. Studies evaluated on only one dataset were not automatically excluded; they were retained when they addressed broader methodological challenges, such as preprocessing sensitivity, calibration, or cross-validation. Conversely, a study was excluded under this criterion if its sole contribution was an incremental performance gain on a specific benchmark, lacking broader relevance to the review’s primary questions.

The initial screening identified 1047 Include records, 578 Unclear records, and 10 Background sources. The working screening plan anticipated further assessment of both Include and Unclear records. In practice, the evidence-focused candidate pool was constructed from the 1047 Include records plus seven records reinstated after a quality-control check of false exclusions, while the 578 Unclear records were not advanced to the same second-stage assessment. This was a deviation from the intended workflow, not a determination that the Unclear records were ineligible. Because the review was conducted sequentially by a single reviewer and these records were not re-adjudicated in the focused stage, the deviation may have introduced selection bias and is treated explicitly as a limitation of the review.

#### 2.6.2. Evidence-Focused Second-Stage Selection

The second stage reviewed 1054 candidate records: the 1047 records initially classified as Include and seven records reinstated after bibliographic and relevance checks. Its purpose was to construct a coherent evidence corpus for the systematic mapping synthesis rather than to catalogue every EEG classifier returned by the broad searches. Accordingly, this stage is interpreted as evidence-focused curation for systematic mapping, not as an exhaustive identification of every publication that might satisfy the broader eligibility criteria.

Priority was given to four evidence domains:

- fragmentation, interoperability, reproducibility;
- datasets and benchmarking;
- cognitive and user variability;
- cross-dataset learning and generalization.

Retention required direct support for at least one review question and a distinct contribution to the mapping framework. Citation count, journal ranking, and the direction of the reported result were not used as selection criteria. Negative findings and failed transfer were eligible when they provided relevant evidence.

Of the 1054 candidate records, 129 were retained: 23 in fragmentation, interoperability and reproducibility; 36 in datasets and benchmarking; 31 in cognitive and user variability; 39 in cross-dataset learning and generalization. The remaining 925 records were excluded for the following documented reasons:

- 449 narrow single-method generalization studies whose contribution was already represented by retained multi-dataset studies, benchmarks, or reviews;
- 180 single-algorithm or application papers without sufficient broader relevance to the review questions;
- 18 peripheral EEG or clinical papers outside the review’s scientific scope;
- 27 dataset-specific papers with limited incremental value after representative paradigms and heterogeneity cases had been retained;
- 222 narrow cognitive-state classifier or application papers with limited evidence on variability, reproducibility or interoperability;
- 28 broad or generic reviews with limited incremental value after the most directly relevant syntheses had been retained;
- one residual duplicate record.

Because judgments about redundancy and incremental contribution were involved, this stage is reported as evidence-focused curation rather than as an objective full-text exclusion process. The resulting 129 publications therefore constitute a curated synthesis corpus rather than an exhaustive eligible population. Counts, percentages, and evidence-map frequencies derived from these publications are descriptive of the retained corpus and should not be interpreted as prevalence estimates for the entire EEG-BCI literature. Screening was sequential and was not independently duplicated; consequently, an inter-rater agreement coefficient was not calculated.

#### 2.6.3. Full-Text Eligibility Confirmation

After the curated 129-publication synthesis corpus had been defined, the full text of every retained publication was obtained and reviewed before extraction and transparency scoring. The reviewed sources comprised 45 publisher or PubMed Central open full texts, 66 publisher versions or author manuscripts, and 18 proceedings papers or author-manuscript versions. Each publication was checked against the general and thematic eligibility criteria, its bibliographic identity, publication type, and assigned evidence domain. All 129 publications remained eligible for the final synthesis.

This full-text step confirmed the eligibility and content of the retained corpus and provided the evidence base for extraction. It did not retrospectively convert either the non-advancement of the 578 Unclear records or the 925 evidence-curation exclusions into conventional full-text eligibility decisions. This distinction is important when interpreting the adapted PRISMA flow and the limitations of the review.

**Figure 1:**
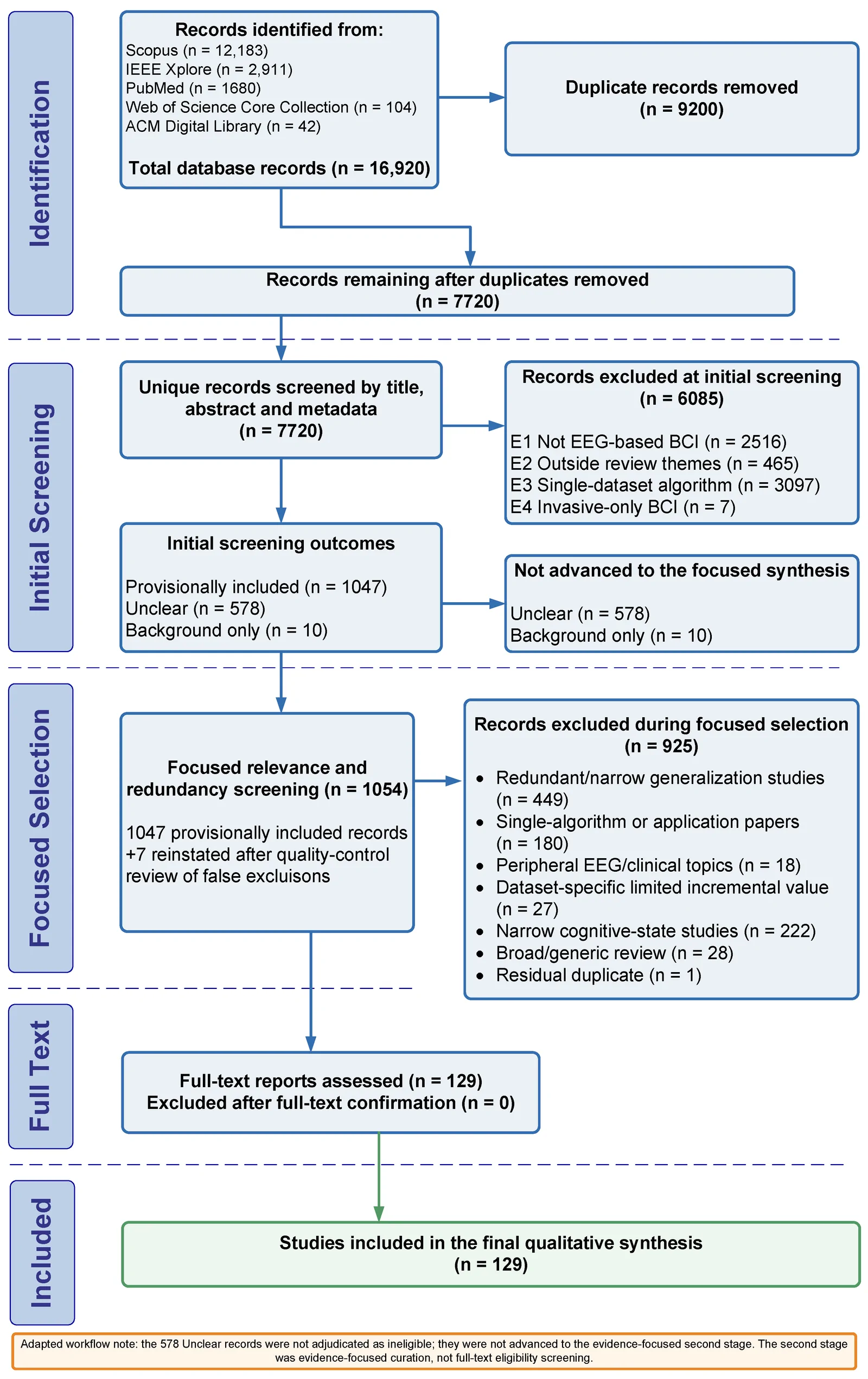
Adapted PRISMA 2020 workflow for study identification, initial screening, evidence-focused selection, full-text confirmation, and inclusion. The 578 records classified as Unclear were not adjudicated as ineligible but were not advanced to the evidence-focused second stage. The 129 retained publications were subsequently assessed in full text, with no exclusions at confirmation. Accordingly, the diagram documents the implemented systematic mapping workflow and the construction of a curated synthesis corpus; it should not be interpreted as a conventional full-text assessment of every record initially classified as Include or Unclear.

Figure 1 reports the actual selection workflow and explicitly separates title, abstract, and metadata screening; evidence-focused selection; full-text eligibility confirmation; and final inclusion. It should be interpreted as an audit trail of the implemented systematic mapping workflow, not as a conventional PRISMA full-text assessment of every record initially classified as Include or Unclear.

The searches yielded 16,920 records. After removal of 9200 duplicates, 7720 unique records were screened. Initial screening excluded 6085 records and identified 1047 provisionally eligible records, 578 Unclear records, and 10 Background sources. Seven records were reinstated after quality-control review of false exclusions, producing an evidence-focused pool of 1054 records. The focused selection excluded 925 records. The full texts of the 129 retained publications were then assessed, with no further exclusions, and all 129 publications entered the final curated synthesis corpus.

### 2.7. Data Extraction

A predefined extraction matrix was used to collect bibliographic, technical, methodological and conceptual information. Final extraction was based on the full texts of all 129 included publications and on supplementary material or linked repositories where available. Publisher records and database metadata were used to confirm bibliographic details, DOI information, publication type, and data or code links; abstracts and database exports were not used as substitutes for full-text evidence. Information not stated explicitly was coded as Not reported rather than inferred.

The full-text audit was performed publication by publication. For each record, the extraction file retained the full-text source status, evidence notes, missing or unclear items, and a confidence flag so that uncertain judgments could be identified and traced during verification.

The confidence flag described confidence in source verification for the extraction, rather than confidence in the scientific validity of a study or certainty of evidence. High confidence was assigned when a publisher or PubMed Central open full text was directly reviewed and the relevant reporting evidence could be verified directly (*n* = 45). Medium confidence was assigned when a publisher-accessible or author-manuscript/proceedings full text was reviewed using conservative coding because the source representation or one or more extraction fields were less directly verifiable (*n* = 84). No Low-confidence records were present in the final 129-publication audit. When information remained missing or ambiguous, the affected item was recorded as Not reported rather than inferred, and the uncertainty was retained in the evidence notes.

The extraction fields were grouped into six categories.

Bibliographic and provenance fields included record identifier, title, authors, year, DOI, URL, database source, search family, document type, study type, full-text source status, coding confidence.

BCI and dataset fields included BCI paradigm, dataset name, public or private status, repository, number of subjects, number of sessions, participant group, and whether the data represented healthy participants, patients, or mixed populations.

Acquisition and representation fields included sampling rate, number of channels, electrode system, file format, event or annotation format, metadata completeness, and any explicitly reported data standard, ontology, common model, or interoperability tool.

Reproducibility fields included preprocessing description, data availability, code or pipeline availability, evaluation protocol, benchmark use, and the main reproducibility issue identified by the publication. Particular attention was paid to preprocessing sensitivity, data leakage, inconsistent train and test partitioning, cross-validation design, and incomplete reporting, because these factors can substantially change EEG decoding results [15,31,33,35,36,37].

Generalization fields captured the specific scope of the analysis: cross-subject, cross-session, or cross-dataset evaluation; alongside the chosen strategy, ranging from transfer learning and domain adaptation to calibration reduction. Cross-subject and cross-session evaluations were coded separately to account for their distinct sources of variability. Cross-dataset evidence was only assigned if independently collected datasets were actively transferred, merged, or aligned within a joint analysis, rather than simply evaluated in parallel but unrelated experiments.

Cognitive and user-variability fields tracked a broad spectrum of human factors, encompassing user-state metrics like mental workload, fatigue, attention, motivation, and broader inter-subject or temporal variations. These terms were retained separately because they are measured differently and may affect BCI performance through distinct mechanisms [38,39,40,41,42,44,47,48,49,51,52,53,54,60,65,66,68].

The matrix also contained two interpretative fields: the main interoperability issue described or implied by the publication, and its relevance to semantic interoperability. These fields distinguished public or technically readable data from data whose task, event, sensor, processing, and participant semantics were sufficiently explicit for cross-dataset reuse.

### 2.8. Coding Framework

The evidence was coded using a multidimensional fragmentation framework. Five principal dimensions were distinguished.

Structural fragmentation covered file formats, folder organization, channel naming, sampling rates, montage differences, coordinate systems, annotation structures, and incompatible data containers.

Semantic fragmentation covered differences or ambiguity in task definitions, event meaning, class labels, trial boundaries, stimulus descriptions, participant state, and the interpretation of BCI paradigms.

Procedural fragmentation covered acquisition protocols, calibration procedures, preprocessing, artifact rejection, feature construction, model-validation design, and reporting practices.

Human and contextual fragmentation covered inter-subject variability, health status, age, fatigue, workload, motivation, attention, session drift, environmental conditions, and laboratory versus real-world use.

Computational fragmentation covered dataset-specific software, unavailable code, incompatible pipeline assumptions, benchmark differences, and models that could not be transferred across datasets or acquisition conditions.

A study could receive more than one code. For example, a multi-session motor-imagery dataset could contribute evidence about structural organization, session variability, and scalable learning. Likewise, a transfer-learning paper could be coded under both computational fragmentation and user variability if its method explicitly addressed inter-subject differences.

Interoperability solutions were coded into five corresponding groups:

1. data organization standards, common models, and ontologies;
2. repositories and openly distributed datasets;
3. reusable software and benchmark frameworks;
4. harmonization, alignment, and dataset-merging methods;
5. learning-based approaches to cross-subject, cross-session, or cross-dataset generalization.

This arrangement prevented software frameworks and transfer-learning algorithms from being treated as equivalent solutions. A benchmark platform may make evaluation more consistent, whereas an ontology addresses meaning and a domain-adaptation method addresses statistical distribution shift. Each solves a different part of the fragmentation problem.

### 2.9. Methodological Transparency Assessment

A ten-item methodological transparency score was applied to support descriptive comparison of reporting practices. One point was assigned for each of the following:

1. the dataset was clearly identified;
2. data availability was stated;
3. the number of subjects and sessions was reported;
4. acquisition parameters were reported;
5. channel or electrode information was provided;
6. preprocessing steps were described;
7. tasks, events, or class labels were described;
8. the evaluation protocol was reported;
9. code or pipeline availability was stated;
10. reproducibility or generalization limitations were discussed.

An item received one point only when it was supported explicitly by the reviewed full text, supplementary material or a linked repository record. No partial points were assigned, and missing or ambiguous information was scored as zero rather than inferred from common practice. The audit file retained the evidence note, missing or unclear criteria, full-text source status, and the confidence for each publication.

The maximum score was 10. Scores of 8–10 were classified as high transparency, 5–7 as moderate, and 0–4 as low. No weighting or applicability adjustment was applied. The same ten criteria were used for dataset papers, empirical studies, benchmarks, reviews, and standards or software publications. Consequently, total scores were interpreted by publication type because several acquisition- or participant-related criteria can be outside the purpose of a review, standard, or conceptual framework.

The ten criteria were deliberately unweighted, so each contributed exactly one point to the total score. Consequently, absence of a code or pipeline availability statement reduced the score by only one point: a publication that satisfied the other nine criteria could still receive 9/10. The median score of 9/10 is therefore compatible with code or pipeline availability being stated in only 20.9% of the retained corpus. The total score summarizes the breadth of reporting across the predefined criteria rather than the openness of research materials.

The score measured reporting transparency, not open-science completeness, methodological validity, study quality, risk of bias, or certainty of evidence. A low score did not imply that the scientific conclusions were invalid, and a high score did not establish that the study design was unbiased or that data and code were openly available. The high, moderate, and low categories were therefore descriptive reporting bands rather than quality grades.

The criteria were informed by recurring concerns in BCI benchmarking and EEG machine-learning literature, including incomplete dataset identification, sensitivity to preprocessing, inconsistent validation, unavailable pipelines, and inadequate discussion of generalization limits [14,15,17,18,19,31,34,35,36,37].

### 2.10. Evidence Synthesis

The evidence was synthesized descriptively and thematically. Meta-analysis was not attempted because the retained publications differed substantially in BCI paradigm, task structure, participant population, dataset size, sensor configuration, preprocessing, validation scheme, outcome measure, as well as learning scenario. Classification accuracy from a within-subject motor-imagery experiment, for example, is not directly comparable with cross-session workload estimation, P300 spelling, or cross-dataset SSVEP transfer.

The quantitative mapping included publication year, study type, BCI paradigm, dataset availability, number of subjects and sessions where reported, cognitive variables, cross-subject or cross-session design, cross-dataset evaluation, transfer-learning use, code availability, and transparency score.

The thematic synthesis followed the five review questions. For each fragmentation dimension, the analysis considered:

- the type of incompatibility reported;
- its consequence for reuse, comparison, or learning;
- the solution proposed in the literature;
- the level at which that solution operated;
- the limitation that remained after the proposed solution was applied.

The final synthesis was organized around four evidence domains: fragmentation/interoperability/reproducibility, datasets/benchmarking, cognitive/user variability, and cross-dataset learning/generalization. Connections between the domains were treated explicitly. Dataset differences were linked to reproducibility problems; user and session variability were examined as data-context variables; interoperability approaches were assessed according to whether they addressed structure, meaning, procedure, or statistical alignment; and scalable-learning studies were evaluated in relation to the heterogeneity they attempted to overcome.

The review did not assume that successful transfer learning demonstrated semantic interoperability. A model may reduce statistical distribution shift while leaving task definitions, event semantics, acquisition context, or preprocessing provenance unresolved. Conversely, a semantically well-described dataset may remain difficult to combine with another dataset because of incompatible sensor layouts or insufficient signal quality. The synthesis therefore distinguished format compatibility, metadata completeness, semantic equivalence, statistical alignment, and practical learnability.

### 2.11. Audit Trail and Reproducibility of the Review

The review materials were maintained in structured workbooks containing the search log, retrieved records, deduplicated screening matrix, controlled vocabularies, second-stage decisions, retained references, full-text transparency assessment, and summary statistics. The search log preserved the exact query, database, search date, result count, and source filename for each search family.

Every retained record was linked to its screening decision and database provenance. The 925 s-stage exclusions remained in the audit workbook together with the recorded curation rationale. The final 129 publications were linked to their full citation, DOI or source URL, full-text status, study type, transparency criteria, total score, confidence flag, and evidence notes.

Bibliographic information was not silently reconstructed when database exports lacked volume, page, conference location, or author details. Such entries were flagged for verification. The same principle was applied to substantive extraction: missing acquisition, metadata, preprocessing, or availability information was coded as Not reported rather than inferred.

The audit trail permits the selection, extraction, and transparency scoring to be inspected and revised. It also records the principal deviations from the intended workflow, including the non-advancement of the 578 Unclear records and the use of an evidence-focused curation stage before full-text verification of the 129 retained publications. Verification of reconstructed Web of Science and ACM search records and final bibliographic formatting remains separate from the eligibility confirmation already completed for the synthesis corpus.

### 2.12. Use of Generative AI Tools

OpenAI ChatGPT (GPT-5.6 Thinking; OpenAI; last accessed 1 August 2026) and the image-generation capability integrated in ChatGPT were used as assistive tools for language editing, consistency checking, and final proofreading. Final eligibility decisions, publication-level scores, scientific interpretations, and figure content remained the author’s responsibility. The author reviewed and revised all AI-assisted outputs and takes full responsibility for the content of the manuscript.

## 3. Results

The results of the systematic mapping review are presented thematically. This section characterizes the public EEG-BCI dataset landscape and its principal forms of technical fragmentation; Section 4 and Section 5 report the mapped findings on cognitive/user variability and reproducibility, respectively, while Section 6, Section 7 and Section 8 develop the interoperability implications and future research directions.

### 3.1. Overview of Public EEG-BCI Datasets

Public EEG-BCI resources offer a rich but highly diverse collection of neural data, rather than a uniform, interchangeable pool. Because these datasets originate from varied paradigms, participant groups, and acquisition systems, and often provide uneven levels of metadata and processing history, their potential for reuse depends as much on understanding their experimental context as on their technical accessibility [1,2,3,4,5,6,7,8,9,10,11,12,13,117,118,119,120,121,122,123,124,125,126,127,128,129,130].

**Figure 2:**
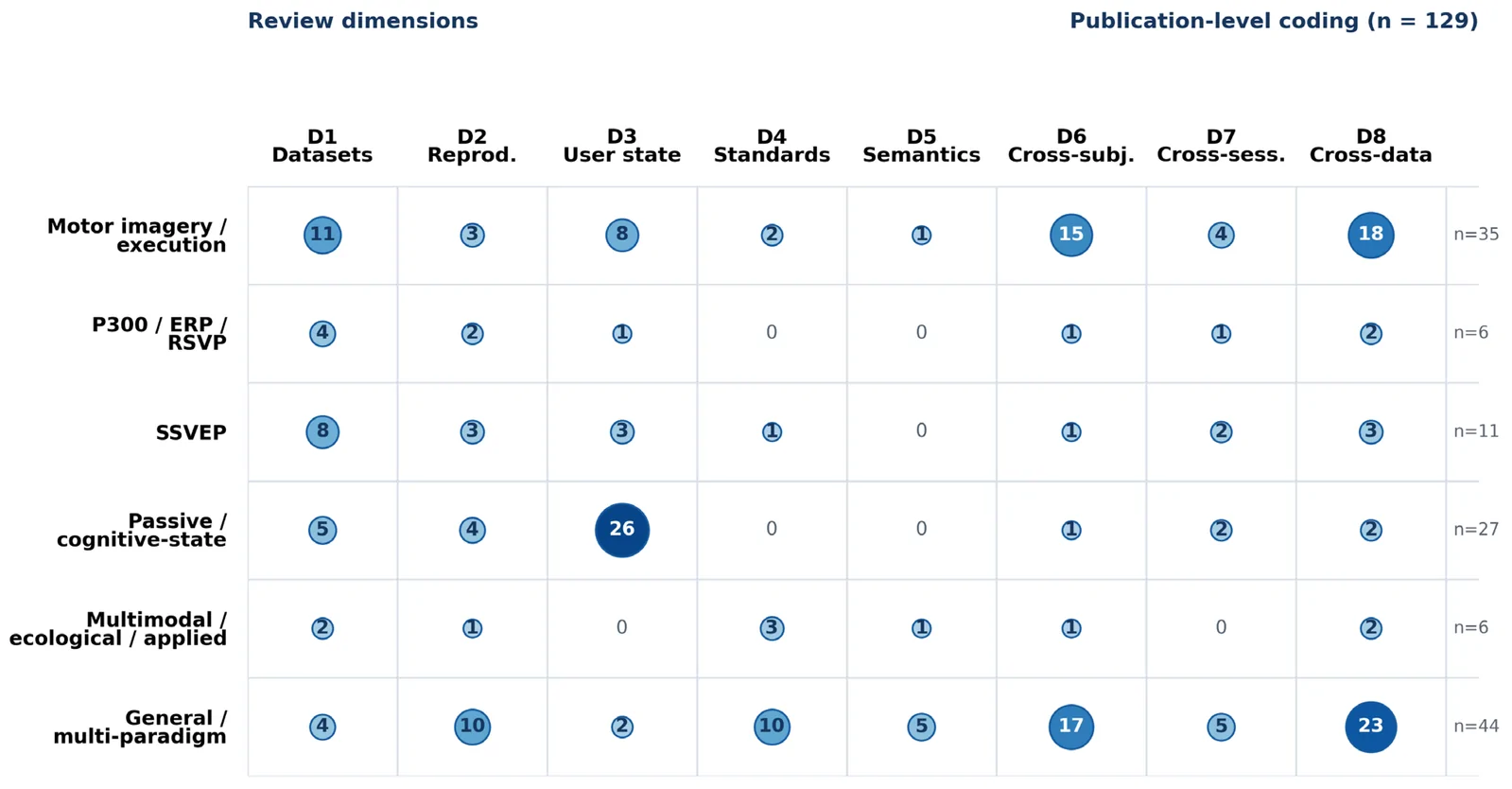
Count-based publication-level evidence map of the curated 129-publication EEG-BCI synthesis corpus. Cell values and bubble areas indicate the number of coded publications in each paradigm-by-dimension cell. Each publication was assigned to one primary paradigm grouping and to one or more review dimensions; counts are therefore non-exclusive across columns. The eight labeled review dimensions (D1-D8) correspond, respectively, to public datasets/resources, reproducibility/benchmarking, cognitive/user variability, interoperability/standards, semantic/context representation, cross-subject transfer, cross-session/calibration, and cross-dataset/scalable learning. The unit of counting is the publication rather than an independent dataset.

Figure 2 presents a count-based evidence map generated directly from publication-level coding of the curated 129-publication synthesis corpus. Each publication was assigned one primary paradigm grouping and one or more review dimensions. The resulting matrix confirms the dominance of motor-imagery and general methodological work in transfer-oriented research, while passive and cognitive-state BCI studies contribute most strongly to the evidence base on user variability.

Because the unit of quantitative coding was the publication, the 129-publication total should not be interpreted as 129 independent EEG-BCI datasets. Of the retained corpus, 22 publications were classified as Dataset/Resource contributions in the extraction matrix: SSVEP (*n* = 7), motor imagery/execution (*n* = 6), P300/ERP/RSVP (*n* = 3), general/multi-paradigm (*n* = 2), passive/cognitive-state (*n* = 2), and multimodal/ecological/applied BCI (*n* = 2). These publications do not necessarily correspond one-to-one to unique datasets because a resource may contain multiple paradigms, while multiple methodological publications may reuse the same public dataset. Dataset diversity is therefore interpreted through resource identity and properties such as participants and sessions, sensor topology, sampling, task/event semantics, and data organization rather than through publication count alone [1,2,3,4,5,6,7,8,9,10,11,12,13,117,118,119,120,121,122,123,124,125,126,127,128,129,130]. Table 1 provides representative examples of these resource-level differences.

### 3.2. Major BCI Paradigms

#### 3.2.1. Motor Imagery and Motor Execution Datasets

Motor imagery (MI) is the most represented paradigm in the public-dataset and transfer-learning literature. Available resources range from controlled recordings to multi-session and multi-day collections designed to expose temporal drift, and several include questionnaires, electrode locations, demographic variables, or out-of-laboratory acquisition [2,3,4,7,9,12,13,125]. Nevertheless, apparently identical left- or right-hand labels may be associated with different parameters: cue timing, imagery instructions, feedback, trial duration, channel montage or calibration. Cross-dataset MI learning therefore combines physiological distribution shift with protocol and semantic differences.

#### 3.2.2. P300 and RSVP Datasets

ERP resources commonly grouped as P300 datasets include materially different tasks. RSVP datasets usually represent rare-target detection in an image stream, whereas speller datasets derive target status from stimulus-selection sequences; collaborative, benchmark, clinical, and multicenter variants introduce further differences [5,6,8,103,122]. A target or non-target label can represent anything from an isolated image or flash to a specific row, column or reconstructed character. Because of this semantic variety, simply standardizing the numeric labels is insufficient. Researchers must also explicitly reconstruct the underlying event timing, stimulus structure, and feedback mechanisms to ensure accurate interpretation [30].

#### 3.2.3. Steady-State Visual Evoked Potential Datasets

SSVEP datasets are defined not only by EEG recordings but also by their frequency and phase coding, display timing, luminance modulation, viewing geometry, and trial design. Public resources span conventional benchmarks, single-channel and wearable systems, hybrid paradigms, broad stimulation ranges, and augmented-reality settings [1,117,118,120,124,128]. The stimulus apparatus is part of the signal-generating system; consequently, equal nominal frequencies do not guarantee equivalent recording conditions when presentation parameters are missing or differ.

#### 3.2.4. Passive, Multimodal, and Ecological BCI Datasets

Passive and ecological BCI datasets generally capture complex user states, such as cognitive workload, fatigue, and engagement. This is done by integrating task manipulations with behavioral or physiological metrics. The ecological validity of these resources is frequently broadened through diverse experimental paradigms, ranging from simulated driving and repeated cognitive tasks to continuous wearable field recordings [10,11,123,126,127,130]. The labels derived from these studies are highly context-dependent. A specific vigilance classification, for instance, holds meaning only when grounded in its original induction task, temporal parameters, and operational environment. In this study, it was retained only if such resources maintained an explicit BCI focus and offered substantive evidence regarding data reuse, system variability, or benchmarking.

### 3.3. Dataset Characteristics

Table 1 compares a selection of representative public resources by outlining key differences in their experimental paradigms, participant structures, acquisition setups, and data formats. Rather than serving as an exhaustive catalogue, this overview highlights the specific dataset characteristics that ultimately determine whether diverse resources can be seamlessly integrated and processed through a unified analytical workflow.

Simply counting available datasets offers a poor proxy for actual interoperable training volume. Beyond using divergent file formats, ranging from BIDS and EDF to custom MATLAB structures, public resources frequently differ in fundamental ways, such as channel layouts, session spacing and event definitions [3,4,5,7,8,10,11,12,26,124]. Multi-paradigm collections facilitate within-participant comparisons but do not create a common prediction unit, while datasets from the same paradigm may remain incompatible because of session or protocol differences. To successfully select data for scalable learning, researchers need to establish explicit compatibility criteria instead of relying on broad paradigm names alone.

### 3.4. Technical Fragmentation

#### 3.4.1. Sensor and Acquisition Representation

Channel count is only the most visible acquisition difference. Electrode subsets, cap layouts, names, references, coordinates, units, amplifiers, trigger paths, and sampling rates all vary across the corpus [1,2,3,4,5,6,7,8,9,10,11,12,13,117,118,119,120,121,122,123,124,125,126,127,128,129,130]. Paradigm-specific apparatus also matters: SSVEP depends on display timing, mobile recordings introduce motion and contact variability, and multimodal studies require synchronization. A common reader can remove a syntactic barrier while leaving the acquisition context unresolved.

#### 3.4.2. Event and Task Semantics

Experimental events are frequently encoded as simple integers or brief labels, leaving their true meaning dependent on external papers, scripts, or repository notes. Consequently, an identical marker might indicate a stimulus onset in one dataset but a derived class in another, while paradigms such as P300 or RSVP may assign target status to completely different levels of the experimental hierarchy [5,8,30]. Any defensible cross-dataset mapping must preserve this broader context, including the interplay between trial structures, participant instructions, and the intended analytical unit. Simply adopting a shared vocabulary can otherwise mask underlying incompatibilities.

#### 3.4.3. Data Organization and Processing Provenance

Datasets are distributed across various processing stages, ranging from raw continuous recordings to highly derived feature-level releases. They are also typically packaged in multiple container formats. Routine operations such as filtering, spatial re-referencing, and artifact removal may have already fundamentally altered the available signals prior to distribution. While frameworks such as EEG-BIDS enhance structural consistency [26], and containerized workflows can preserve executable processing [69], neither solution can reconstruct acquisition or preprocessing choices that were simply left undocumented.

#### 3.4.4. Participant, Session, and Context Metadata

Participant and session metadata can vary significantly, ranging from sparse demographic details to comprehensive profiles that capture health status, task experience, and complex environmental or cognitive states [7,9,10,11,12,13,126,130]. Because these variables can act as prediction targets, experimental confounders, or strict boundaries for cross-dataset transfer, establishing interoperability requires highly transparent data tracking. Researchers must explicitly record the provenance and transparently acknowledge the absence of stable participant traits, session-specific conditions, and fluctuating user states. Because this fragmentation is so deeply entangled across neurophysiological signals, experimental semantics, and human contexts, achieving meaningful interoperability extends far beyond simple file conversion.

## 4. Cognitive Variability and Human Factors

EEG-BCI observations are inherently tied to both the user and the specific conditions of the task. Physiological variables such as fatigue and health status, alongside environmental and cognitive factors such as workload and motivation, all influence the measured neural signals [38,39,40,41,42,43,44,45,46,47,48,49,50,51,52,53,54,55,56,57,58,59,60,61,62,63,64,65,66,67,68]. Whether these elements act as the primary prediction target in a passive BCI or operate as unmeasured variables in active systems, they fundamentally shape the meaning of the dataset.

### 4.1. Inter-Subject Variability

Large between-user differences are recurrent in sensorimotor and other EEG-BCI paradigms [38,39,40]. A diverse array of factors, ranging from anatomical and clinical traits to signal quality and user strategy, drives this variability. Yet, public datasets document these elements inconsistently [9,65,66,67,68,125]. While alignment and domain-adaptation algorithms can successfully minimize statistical distribution shifts, they cannot identify the specific physiological or contextual drivers that originally caused those differences. To properly account for this, cross-subject evaluations must move beyond simply reporting pooled accuracies and instead explicitly detail individual participant-level performance alongside relevant metadata descriptors.

### 4.2. Session-to-Session Variability

Repeated recordings naturally drift over time due to a combination of physical variations, such as minor shifts in electrode placement or impedance, and human factors, such as task learning, fatigue, and unrecorded cognitive changes. Although multi-day MI and passive-BCI datasets make this temporal generalization observable [7,10,12,39,60,62,121], accurately interpreting these shifts requires detailed metadata encompassing session chronology, physical cap adjustments, and individual training histories. Calibration should not be treated as a neutral remedy; any comparative analysis must explicitly disclose how much target-session data and label information were used and which model parameters were updated [79,80].

### 4.3. Mental Fatigue, Cognitive Load, and Related User States

#### 4.3.1. Mental Fatigue

Mental fatigue has been extensively investigated across various active and adaptive BCI paradigms, ranging from sustained-attention tasks to standard event-related potential and motor imagery protocols [41,42,43,44,45,46,47,48,49,50,51]. Rather than converging on a single universal marker, studies employ a wide array of physiological and behavioral metrics. Consequently, a given fatigue label might indicate an experimentally induced condition, a purely subjective self-assessment, or a complex model-derived state. To enable meaningful cross-study comparisons, researchers must ensure that their specific measurement methods, temporal resolutions, and exact relationships to the underlying BCI task remain entirely explicit.

#### 4.3.2. Cognitive Load, Workload, Attention, and Vigilance

Workload and vigilance are prominent passive-BCI targets in laboratory and operational tasks [52,53,54,55,56,57,58,59,60,61,62,63,64,126]. The labels used to quantify these states vary widely, originating from sources such as subjective ratings, behavioral metrics, or dynamic system states. The specific temporal epoching and data aggregation rules applied to these labels can significantly alter the underlying prediction problem. While domain adaptation techniques effectively reduce statistical feature shifts, they cannot confirm that workload constructs induced by entirely different experimental tasks are equivalent. To ensure clarity, cross-study transfer evaluations must explicitly report their operational definitions and reference measures rather than relying solely on generic class names.

#### 4.3.3. Motivation, Emotion, Experience, and Ecological Context

Although cognitive factors such as motivation, mental state, and individual training history significantly impact performance, they remain far less consistently documented than either fatigue or workload [65,66,67,68,115]. As research moves toward more ecological settings, including wearable or vehicle-based platforms, these settings introduce new physical and behavioral constraints affecting factors ranging from sensory load to sensor contact quality [13,126,130]. Rather than viewing these real-world conditions as mere deviations from a controlled laboratory norm, researchers should treat them as fundamental boundaries that define both the system’s intended use context and its overall capacity for generalization.

Table 2 summarizes the principal human and cognitive variables, how they are represented, and their implications when datasets are compared or combined.

### 4.4. Impact on Reproducibility and Generalization

Human variability fundamentally impacts how experiments are measured, evaluated, and ultimately replicated. Even when paradigms appear identical, they frequently diverge in their underlying cognitive demands, training protocols, or feedback mechanisms. Critical user-state variables are often omitted altogether or recorded using completely incompatible instruments and temporal scales. While statistical alignment and calibration reduction techniques can improve cross-study transfer [23,56,62,74,79,80,109,110,111,112,113,114,115,116], they cannot bridge these fundamental gaps in construct meaning. To create truly reusable data representations, researchers must clearly separate stable participant traits from fluctuating, session-specific states, documenting exactly how each factor was measured while transparently preserving any uncertain information. Thus, user variability remains a complex, intrinsic feature of BCI data rather than a simple nuisance variable that can be erased through algorithmic adaptation.

## 5. Reproducibility Challenges in EEG-Based BCI

Vulnerabilities can emerge at any stage of the research workflow. The limitations of initial data acquisition dictate what information can ultimately be recovered, while subsequent preprocessing choices fundamentally alter the signal representation. Thus, experimental protocol decisions define the core task, and the chosen validation strategies determine the specific claims that can be reliably tested [14,15,31,32,33,34,35,36,37].

### 5.1. Technical Reproducibility: Acquisition and Preprocessing

EEG outcomes are highly susceptible to a wide array of preprocessing choices, ranging from filtering and spatial referencing to artifact rejection and normalization techniques [31,33]. Because of this sensitivity, achieving true reproduction demands far more than a generic statement that standard preprocessing was applied. Researchers must transparently document their entire analytical pipeline, explicitly detailing the precise sequence of operations, exact software parameters, and the specific rationale for any rejected observations. While open access to raw data remains highly valuable, it cannot overcome the absence of critical hardware specifications such as channel coordinates or amplifier trigger latencies. Ultimately, adopting structured metadata schemas alongside executable workflows offers a practical way to address these complementary aspects of the reproducibility challenge [26,69].

### 5.2. Experimental Reproducibility: Paradigms, Sessions, and Calibration

Broad paradigm labels such as MI, P300, or SSVEP often mask substantial variations in how tasks are actually implemented, encompassing underlying differences in participant instructions, stimulus timing, and class construction [30,115]. Logistical variations, such as the spacing between recording sessions, physical cap replacements, and intermittent protocol adjustments, are also fundamental components of the broader experimental design. Consequently, claims regarding reduced or zero calibration can only be meaningfully evaluated when researchers explicitly outline their validation procedures, detailing exactly how much target data and label information were accessed and the rules governing parameter updates and model stopping [37,79,80].

### 5.3. Computational Reproducibility: Code, Evaluation, and Software Environments

Providing a public repository link is a necessary but insufficient step for true analytical reproduction. To fully recover an analysis, the documentation must capture the exact computational environment, including specific code revisions, hardware assumptions, software dependencies, and precise modeling details such as random seeds and data-splitting logic [15,35,36]. Because EEG observations exhibit strong hierarchical dependencies, employing simple random trial splits risks leaking session- or participant-specific information directly into the test set. Robust evaluations must explicitly define their cross-validation strategies, detailing the specific partitioning unit, how preprocessing was safely isolated within training folds, and exactly how individual participants and datasets were handled [37].

### 5.4. Benchmarking and Cross-Study Comparability

MOABB and related frameworks improve comparability through common interfaces, pipelines, and evaluation conventions [14,17,18,19], and robustness benchmarks extend assessment beyond mean accuracy [20]. Despite these standardized frameworks, performance outcomes remain highly sensitive to underlying experimental conditions. Consequently, achieving a genuinely reproducible benchmark requires researchers to fix and transparently report the entire experimental configuration. Table 3 summarizes recurrent failure sources and the minimum controls needed to address them.

**Figure 3:**
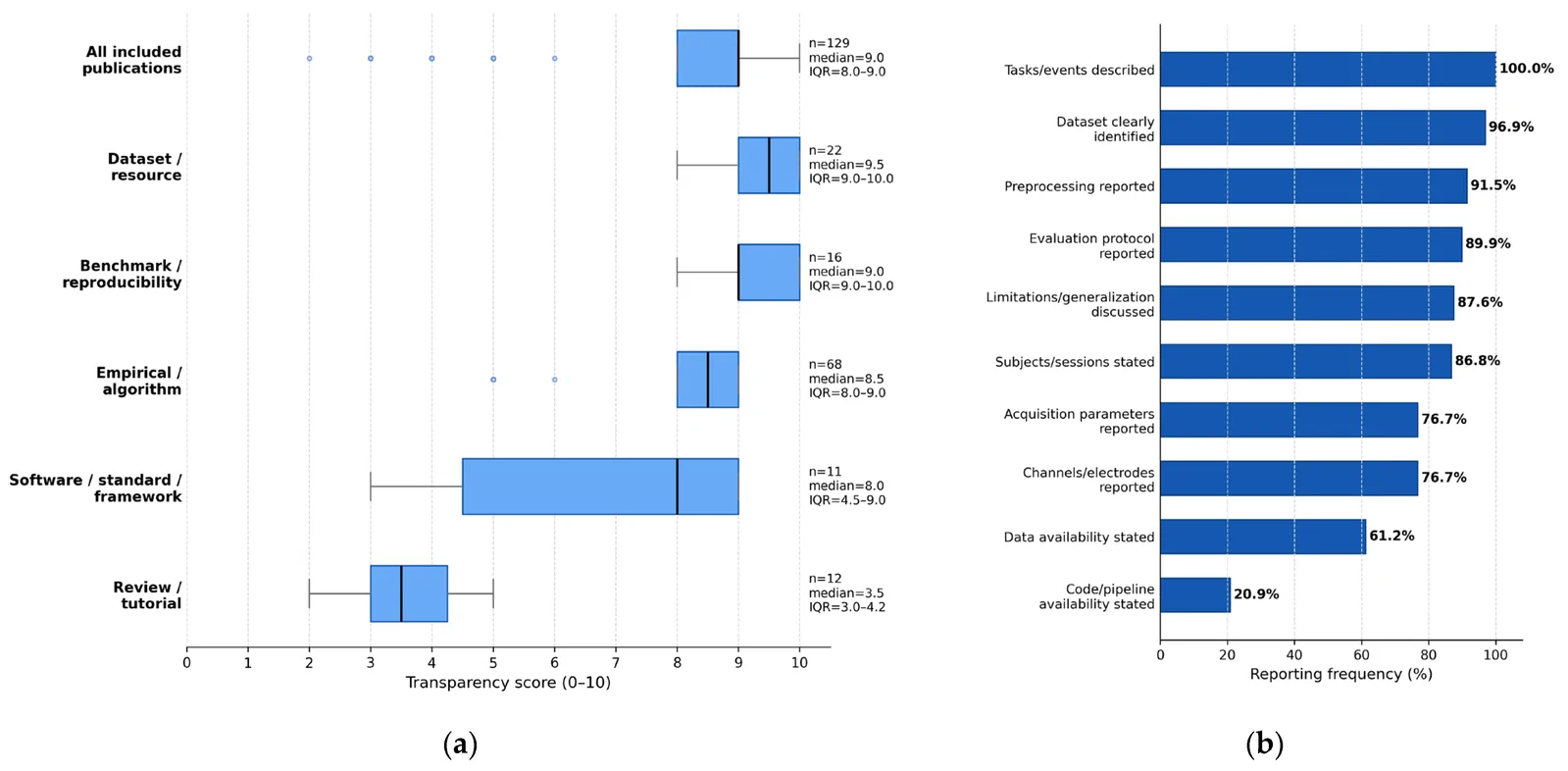
Methodological transparency profile derived from the completed full-text audit of the curated 129-publication synthesis corpus. (**a**) Distribution of total transparency scores for the full corpus and five publication types. Boxes denote the interquartile range, vertical lines indicate the median, and whiskers show the observed range. Blue circles in panel (**a**) denote individual observations lying beyond the boxplot whiskers (more than 1.5 × IQR below the first quartile or above the third quartile). (**b**) Percentage of publications satisfying each of the ten unweighted transparency criteria. Because the criteria are unweighted and several acquisition-oriented items are less applicable to reviews, tutorials, and some standards/framework papers, total scores should be interpreted as reporting breadth within publication type rather than as study quality or risk of bias.

Within the curated 129-publication synthesis corpus, the ten-item transparency assessment was generated directly from the completed full-text scoring matrix (Figure 3). Scores were high for 100 publications, moderate for 17, and low for 12; the median was 9/10 (interquartile range 8–9).

Within the retained corpus, task/event descriptions (100.0%), dataset identification (96.9%), preprocessing (91.5%), and evaluation protocols (89.9%) were commonly reported. Data availability was stated in 61.2% of publications and code or pipeline availability in only 20.9%. The apparently high median score remains compatible with low code availability because the ten criteria are unweighted: absence of a code/pipeline statement reduces the total by only one point. Total scores were also strongly publication-type dependent; accordingly, the measure should be read as reporting transparency within this curated corpus, not as complete openness, methodological quality, or risk of bias.

### 5.5. Reproducibility as a Prerequisite for Interoperability

Simply achieving repeatable execution does not automatically render two datasets comparable. Meaningful cross-study reuse demands that underlying experimental constructs and sensor contexts are carefully aligned, while true semantic interoperability relies on either explicitly defined equivalences or transparently documented transformations. Although tools such as EEG-BIDS, shared repositories, and standardized benchmark interfaces offer crucial structural foundations [18,19,26,27,29,69,70,71,72,73], a dataset’s core scientific meaning must always remain firmly tethered to its original provenance. Building on this, the subsequent section explores how effectively current methodologies address these different interoperability layers and identifies where critical gaps persist.

**Figure 4:**
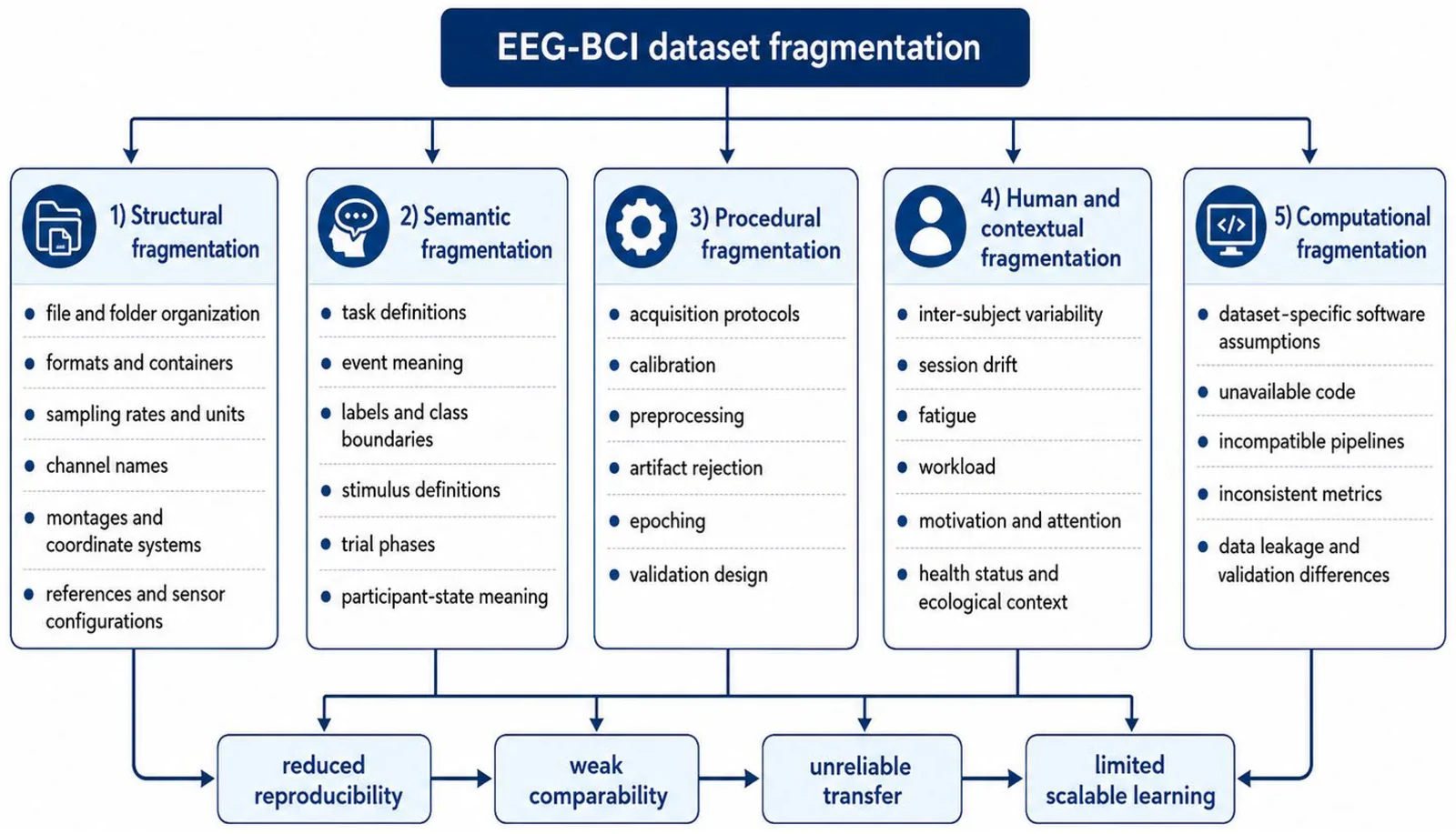
Taxonomy of EEG-BCI dataset fragmentation. Five interdependent dimensions (structural, semantic, procedural, human and contextual, and computational) constrain dataset reuse. Their combined consequences are expressed as reduced reproducibility, weak comparability, unreliable transfer, and limited scalable learning. The categories are non-exclusive and may co-occur within the same dataset or study.

To visually summarize these challenges, Figure 4 synthesizes the various dimensions of fragmentation discussed throughout Section 3, Section 4 and Section 5. It shows how structural, semantic, and procedural discrepancies, alongside broader human and computational factors, interact and compound, ultimately degrading reproducibility and restricting the potential for scalable, cross-dataset learning.

## 6. Existing Interoperability and Standardization Approaches

Existing initiatives address different layers of the BCI data lifecycle. Their contributions are complementary: acquisition platforms coordinate streams and experiments; standards organize stored data; models and ontologies describe concepts; benchmarks normalize access and evaluation; and harmonization or transfer methods align numerical representations. Treating these mechanisms as interchangeable obscures the remaining semantic gap.

### 6.1. Acquisition-Time Interoperability and Experimental Platforms

Lab Streaming Layer supports synchronized multimodal streams and markers during acquisition [131], while BCI2000 and OpenViBE provide established experimental environments [132,133]. MetaBCI and BrainFusion extend reusable acquisition, processing, and deployment workflows [71,72]. These tools improve timing and procedural consistency, but their internal event labels and platform-specific representations do not automatically make independently stored datasets semantically comparable.

### 6.2. Dataset Organization, Metadata, and Processing Provenance

EEG-BIDS provides a major structural basis for participant, session, channel, event, and sidecar metadata [26]. Containerized workflows preserve software environments and transformations [69]. Their limitation is not lack of value but scope: a well-organized directory or executable pipeline cannot reconstruct missing task meaning, participant state, or undocumented preprocessing. Organization and provenance are prerequisites for, rather than substitutes for, semantic interoperability.

### 6.3. Common Models, Ontologies, and Standardization Activities

BCI ontologies and the IEEE P2731 functional model seek consistent descriptions of systems, data elements, and context [27,29,70]. They make relationships machine-readable and can reduce terminological ambiguity. However, practical use across heterogeneous public datasets remains limited, and many legacy event structures or user-state variables cannot be mapped without local interpretation. Conformance must therefore allow partial, uncertain, and versioned mappings.

### 6.4. Benchmarking Frameworks and Dataset Interfaces

MOABB and associated libraries demonstrate the practical value of common loaders, pipelines, and evaluation conventions across public datasets [14,17,18,19]. They strengthen reproducibility and expose dataset-dependent performance, but successful loading does not prove semantic equivalence. Benchmark interfaces generally normalize computational access after dataset-specific interpretation has already occurred, and they cover only the datasets and paradigms for which wrappers exist.

### 6.5. Structural Harmonization and Statistical Alignment

Post-acquisition methods address heterogeneous channel spaces, references, sampling, labels, and distributions through interpolation, dimensional alignment, normalization, transfer learning, and domain adaptation [23,28,30,32,73,75,76,77,78,79,80,81,82,83,84,85,86,87,88,89,90,91,92,93,94,95,96,97,98,99,100,101,102,103,104,105,106,107,108,109,110,111,112,113]. These operations can enable joint processing but alter the representation available to the learner. Their assumptions, fitted parameters, information loss, and source identity should remain explicit; statistical alignment should not be interpreted as proof of semantic compatibility.

### 6.6. Comparative Assessment and Remaining Limitations

Table 4 compares the principal interoperability layers, their strengths, and their remaining limitations.

Conformance verification is therefore a critical missing layer. Declaring a dataset to be EEG-BIDS organized, or making it loadable through a benchmark interface such as MOABB, should not be treated as evidence that all metadata needed for a given analysis are complete or semantically consistent [18,19,26]. A practical approach is to pair standards and interfaces with machine-readable conformance profiles that check required fields, controlled vocabularies, channel and event mappings, version identifiers, and processing provenance, while separately reporting optional or analysis-dependent metadata. Validation should return a structured completeness report rather than only a binary compliant/non-compliant label, because legacy datasets may satisfy structural requirements while remaining semantically incomplete [27,29,70]. Adoption can be encouraged without making unrestricted data release mandatory: repositories can perform automated validation at deposition, journals can request persistent dataset identifiers and validated metadata reports, benchmark libraries can expose dataset-specific mapping assumptions, and funders can recognize reusable metadata and provenance as research outputs. A staged minimum/extended metadata profile would also reduce documentation burden for smaller laboratories while preserving a transparent path toward richer interoperability.

The field does not lack interoperability mechanisms; it lacks a formal connection among them. Streaming and platforms address acquisition, BIDS addresses organization, models and ontologies address description, benchmark frameworks address access and evaluation, and adaptation methods address distribution shift. Scalable cross-dataset research requires these layers to remain linked through dataset identity, signal and sensor structure, task/event meaning, participant and session context, preprocessing provenance, and explicit compatibility decisions. This motivates the representation-oriented synthesis in Section 7.

## 7. Toward Semantic Interoperability and Scalable Learning

True EEG-BCI interoperability extends far beyond simply adopting a shared file format, a common data loader, or a specific alignment algorithm. Instead, the core scientific challenge lies in determining whether observations across different studies hold comparable meaning for a given analysis and, when they diverge, whether a documented transformation remains methodologically sound. A semantic layer should not aim to erase inherent dataset heterogeneity. It must expose these underlying differences clearly enough for researchers to accurately evaluate potential equivalences, necessary transformations, or strict incompatibilities.

### 7.1. Semantic Interoperability as an Analysis-Dependent Property

While syntactic and structural interoperability ensure that basic files can be parsed and experimental variables systematically organized, achieving true semantic interoperability relies on consistent conceptual mapping [26,27,29,70]. Establishing data equivalence is inherently tied to the specific analytical goals of a study. For example, two motor imagery datasets might support a general sensorimotor-rhythm analysis but fail to align on a precise classification problem, just as P300 datasets might use identical target labels yet diverge entirely in their stimulus timing [30]. Thus, researchers should explicitly categorize dataset components into one of three distinct states: practically equivalent, transformable (with acknowledged information loss), or fundamentally incompatible due to insufficient documentation.

### 7.2. Representation Requirements for Heterogeneous EEG-BCI Data

A robust data representation must weave together multiple interconnected layers, including physical sensor configurations, experimental semantics, broader cognitive states and preprocessing histories. These elements are highly relational rather than independent. For instance, a given channel value inherently depends on its specific reference point, just as an event label relies entirely on the surrounding trial structure. Because of these deep dependencies, maintaining persistent data identities, clear versioning, and explicitly documenting missing information are fundamental scientific requirements, particularly for complex user-state variables that remain so unevenly documented across the literature [38,39,40,41,42,43,44,45,46,47,48,49,50,51,52,53,54,55,56,57,58,59,60,61,62,63,64,65,66,67,68].

In practical terms, the semantic layer proposed here should not be conceived as a replacement ontology or a new universal data format. A more feasible starting point is an extensible, machine-actionable interoperability profile that links existing data-organization schemes and BCI conceptual models [26,27,29,70]. Its minimum representation should combine persistent dataset and signal identities with versioning; sensor and channel topology, reference and acquisition context; task, event, label and trial semantics; participant, session and user-state descriptors; preprocessing and transformation provenance; and explicit mapping assertions that classify components as equivalent, transformable, uncertain, or incompatible for a specified analysis. EEG-BIDS can provide part of the structural metadata, while existing BCI models and ontological representations can contribute conceptual vocabularies; the proposed layer would connect these resources through versioned mappings rather than replace them. An initial implementation should therefore begin with a limited interoperability profile for one or two well-documented paradigms, map a small set of public datasets, preserve missing and uncertain fields explicitly, and validate the resulting correspondences before broader cross-paradigm extension.

### 7.3. Provenance-Aware Harmonization and Partial Compatibility

Data harmonization frequently involves a wide spectrum of transformations, ranging from spatial and temporal adjustments, such as resampling or re-referencing, to more complex label aggregation and statistical alignment [28,30,32,73]. To ensure reproducibility, every applied operation must retain a clear lineage to the native data, explicitly documenting the specific mapping rules, fitted parameters, and any information discarded during the process. Researchers must distinguish between purely reversible modifications, such as unambiguous renaming, and destructive changes, such as interpolation or class collapse, which inherently introduce statistical estimates or erase original distinctions. Missing metadata should therefore constrain the analyses that are permitted rather than being silently filled with assumed defaults. Together, these tracking requirements establish the operational foundation for the semantic representations discussed below.

Legacy datasets with incomplete metadata should not be treated uniformly as unusable. Some missing context can be retrospectively recovered from acquisition headers, device or configuration files, stimulus scripts, repository records, supplementary materials, and the original publication. Such recovery must remain provenance-aware: each recovered field should distinguish information explicitly reported in the dataset from information reconstructed from associated artifacts, inferred from indirect evidence, or still unknown. Reconstructed or inferred values should retain their source and confidence and should never silently replace missing native metadata. Dataset admissibility should therefore remain analysis-dependent. A legacy dataset can be retained for analyses whose required compatibility conditions are supported by the available evidence, whereas analyses that depend on unresolved channel references, event semantics, task definitions, participant context, or preprocessing history should be restricted or flagged as uncertain rather than forcing a complete mapping. This approach preserves useful historical data without equating recoverability with semantic equivalence.

### 7.4. Metadata-Aware Cross-Dataset Learning

Transfer and domain-adaptation methods usually operate directly on statistical data structures, such as signal features, covariance structures, or latent representations [21,22,23,24,25,75,76,77,78,79,80,81,82,83,84,85,86,87,88,89,90,91,92,93,94,95,96,97,98,99,100,101,102,103,104,105,106,107,108,109,110,111,112,113]. Semantic metadata serves as a complementary layer. By explicitly defining admissible data sources, potential confounders, and permissible transformations prior to any algorithmic adaptation, this metadata provides critical context that purely data-driven methods lack. Robust evaluations of these techniques must maintain strict dataset boundaries, distinctly reporting performance metrics across individual subjects, discrete sessions, and entirely external datasets rather than relying on generalized pooled results [14,17,18,19]. Although integrating richer metadata theoretically enhances source selection, this assumption remains a hypothesis that demands rigorous empirical validation through targeted ablation studies and deliberately mismatched control scenarios.

Beyond offline cross-dataset learning, the same metadata layer can support adaptive BCI operation by constraining which source models, calibration pools, and preprocessing mappings are admissible for the current user and session state. Session history, fatigue or workload descriptors, montage compatibility, and calibration provenance can therefore serve as contextual variables when selecting a pretrained model or deciding whether and how adaptation is justified [45,50,54,79,80,102,103,111]. The semantic layer itself would not perform online learning; rather, it would expose machine-actionable context and compatibility conditions to an adaptive controller or learning algorithm. This separation is important because adaptation toward a statistically similar but semantically incompatible source may improve apparent fit while undermining interpretation and reproducibility. A practical evaluation should therefore compare metadata-aware and metadata-blind source/model selection while reporting both predictive performance and the compatibility rationale behind each accepted, transformed, or rejected source.

### 7.5. FAIR Principles and Reusable BCI Research Objects

The FAIR principles provide a useful stewardship framework [134], but public access is not equivalent to reusability. A BCI research object should bind data to protocol, metadata, code, environment, versions, licenses, and derivatives, while recording restrictions and uncertainty. General FAIR principles do not define P300 targets, MI cue timing, montage coordinates, or fatigue measures; domain structures such as EEG-BIDS and BCI conceptual models remain necessary [26,27,29].

### 7.6. Scientific Scope and Limits of the Proposed Direction

**Figure 5:**
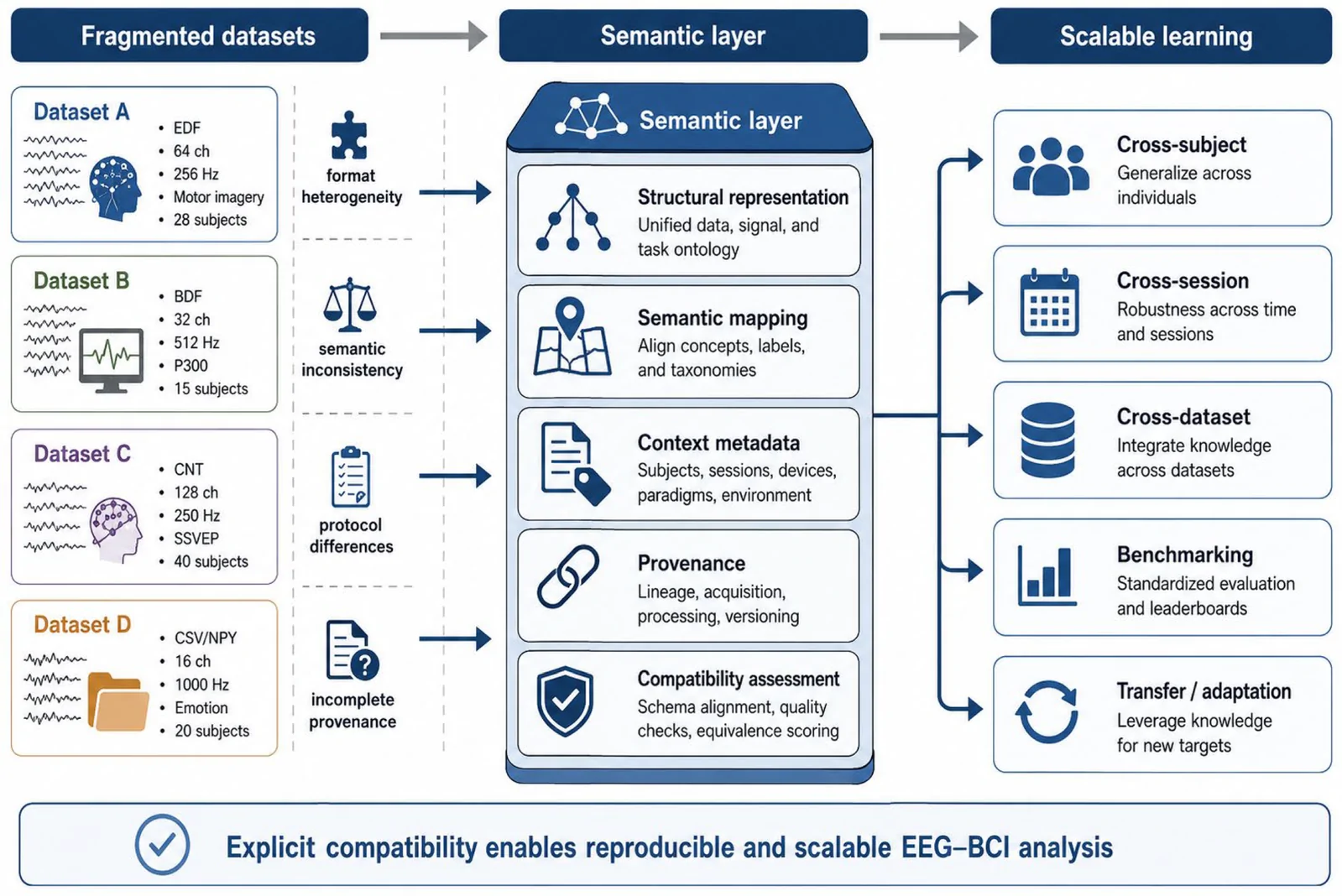
From fragmented datasets to scalable learning through a semantic layer. Heterogeneous EEG-BCI datasets enter a representation layer that integrates structural information, semantic mappings, contextual metadata, provenance, and compatibility assessment. This layer supports cross-subject, cross-session, and cross-dataset learning, reproducible benchmarking, and transfer or adaptation. Dataset labels and numerical values shown in the left panel are illustrative.

The proposed direction is designed to complement, rather than replace, the existing ecosystem of data standards, repositories, and machine learning platforms. The proposed approach does not assume that all legacy datasets can be perfectly reconstructed, nor does it suggest that inherent physiological variability can be entirely normalized or that semantic alignment will automatically guarantee improved model accuracy. Instead, its value lies in streamlining manual data interpretation and establishing highly auditable, reproducible mapping processes. The framework’s success should be measured by how clearly researchers can attribute performance differences and transparently report any uncertain or failed data alignments. To illustrate this, Figure 5 outlines the representation-oriented pathway proposed throughout this review, demonstrating how an explicit semantic layer can effectively bridge the gap between highly fragmented datasets and scalable machine learning applications.

## 8. Future Research Directions

Based on the current evidence, future research should prioritize an agenda that bridges formal data compatibility and auditable transformations with rich contextual representation and metadata-aware machine learning. It should not attempt to engineer yet another universal file format, but rather to establish testable, community-validated rules. These guidelines will provide a rigorous framework for determining exactly when heterogeneous EEG-BCI datasets can be safely compared or effectively leveraged for model training.

### 8.1. Formal Models of Compatibility and Semantic Equivalence

Data compatibility should not be treated as an absolute metric. It must be defined relative to specific analytical targets, factoring in both allowable transformations and acceptable thresholds for information loss. To achieve this, formal representation models must capture the full spectrum of exact, approximate, and unknown relationships, bridging structural variables such as channels and task events with broader human and temporal factors such as participants, discrete sessions, and derived states [26,27,29,30]. Validation protocols must benchmark these mappings against independent expert annotations, ensuring that combined datasets genuinely support the intended analysis without artificially masking underlying methodological disagreements.

A practical gold-standard compatibility set should therefore be constructed as a versioned collection of expert-verified dataset-component pairs rather than as a single canonical dataset. Here, “gold standard” would denote a reference mapping benchmark, not a claim that one dataset, ontology, or vocabulary defines universal semantic truth. The set should include positive cases judged equivalent for a specified analysis; transformable cases accompanied by the required transformation and known information loss; ambiguous cases for which equivalence cannot be established from the available metadata; and negative cases that are deliberately incompatible. Each case should preserve the native metadata and provenance, the intended analysis, the expert rationale, and the expected mapping outcome. Validation should report inter-expert agreement, conformance with the declared mapping rules, reproducibility of any transformation, preservation of analysis-relevant information in downstream tests, and the ability to reject invalid mappings rather than rewarding only successful harmonization. A small benchmark of this kind, spanning several well-documented datasets within one paradigm before extension across paradigms, would provide a reproducible reference for evaluating both manual and automated semantic-mapping procedures [26,27,29,30].

### 8.2. Machine-Actionable Provenance and Auditable Harmonization

Cross-dataset workflows must establish a comprehensive, machine-actionable provenance that captures specific data transformations, including resampling, channel modifications, and artifact handling, together with core computational details such as fitted parameters, software versions, and overarching parent–child data links [28,30,32,69,73]. Automated mapping can reduce manual work, but inferred correspondences must retain confidence and be reviewable. Reproduction should regenerate a derivative from native data and expose where the transformation was irreversible.

### 8.3. Cognitive- and Context-Aware Dataset Representation

To ensure accurate data interpretation, researchers must clearly separate stable participant traits from localized session conditions and fluctuating cognitive states, carefully documenting each factor, including its measurement method, temporal validity, and any missing information [38,39,40,41,42,43,44,45,46,47,48,49,50,51,52,53,54,55,56,57,58,59,60,61,62,63,64,65,66,67,68]. Future studies must critically evaluate which of these contextual variables genuinely enhance model transfer and analysis, distinguishing them from those that merely inflate data collection burdens without offering tangible analytical benefits. The objective is not to compile an exhaustive psychometric profile for every subject, but to isolate the minimum essential context required to support a specifically defined scientific use.

### 8.4. Compatibility-Aware Transfer and Source Selection

Source selection should combine statistical similarity with constraints on task semantics, class definitions, sensor coverage, session design, participant population, and preprocessing lineage [21,22,23,24,25,75,76,77,78,79,80,81,82,83,84,85,86,87,88,89,90,91,92,93,94,95,96,97,98,99,100,101,102,103,104,105,106,107,108,109,110,111,112,113,114,115,116]. Experiments should compare alignment alone, semantic filtering, their combination, and deliberately mismatched sources. This would distinguish negative transfer caused by incompatibility from failure of the learning method.

### 8.5. Cross-Dataset Benchmarks for Scalable Learning

Future benchmarks should preserve dataset identity, use participant- and session-safe partitions, and include leave-one-dataset-out or external-dataset tests [14,15,16,17,18,19,20]. Results should be reported by dataset and subgroup before aggregation, with uncertainty and failed mappings retained. Large-scale EEG models additionally require transparent pretraining inventories, duplicate detection, separation of training and evaluation sources, and tests of statistically and semantically defined distribution shifts.

Recent large-scale EEG models have begun to address channel-topology mismatch at the representation level rather than by requiring every dataset to share one canonical montage. BIOT tokenizes each channel separately and combines channel and relative-position embeddings, allowing cross-data learning when channel sets differ or channels are missing [135]. LUNA instead compresses multichannel EEG into a fixed-size, topology-agnostic latent representation using learned queries and cross-attention, thereby decoupling downstream computation from the original number and arrangement of electrodes [136]. REVE uses a four-dimensional positional encoding to represent electrode location and time, enabling pretraining and downstream use across arbitrary electrode arrangements; its large-scale pretraining spans 92 EEG datasets and 25,000 subjects [137]. These approaches substantially reduce structural and computational barriers caused by heterogeneous montages, but they do not by themselves establish semantic equivalence between channels, references, task definitions, participant contexts, or preprocessing histories. Accordingly, topology-robust representation learning should be treated as complementary to explicit metadata, provenance, and analysis-dependent compatibility assessment. These recent foundation-model examples are discussed contextually and were not added retrospectively to the quantitative 129-publication coding.

### 8.6. Community Adoption, Governance, and Staged Validation

A semantic framework will matter only if it connects with established acquisition platforms, EEG-BIDS, BCI models, repositories, benchmark libraries, and reproducible environments [18,26,27,29,69,131,132,133,134]. Adoption requires versioning, extension rules, validation tools, licenses, persistent identifiers, and governance for ambiguous mappings. Validation should proceed from several datasets within one paradigm to multiple sites and sensor systems, and only then to cross-paradigm claims.

Implementation should proceed in three stages. Immediate work should formalize analysis-dependent compatibility relations, machine-actionable provenance, validation tools, and expert-verified mapping tests on a small number of well-documented datasets within one paradigm. The next stage should evaluate metadata- and context-aware source selection across multiple sites, acquisition devices, and participant or session conditions while retaining dataset identity and uncertainty. Only after these elements are validated should the field pursue broad cross-paradigm interoperability and community-scale automated integration. This ordering treats coverage as an outcome of validated compatibility rather than as the primary objective.

Open science should not be equated with mandatory unrestricted release of all EEG data or code. Consent restrictions, participant privacy, clinical or otherwise sensitive information, intellectual-property and licensing constraints, and the computational and documentation burden on under-resourced laboratories can legitimately limit what is deposited publicly. A practical governance model should therefore define a minimum mandatory metadata and provenance profile, permit controlled-access data and optional extensions, and require justified exceptions when full release is not possible. Journals and funders could reinforce this through persistent dataset identifiers, structured Data Availability Statements, repository deposition, machine-readable and, where feasible, validated metadata, and executable environments or code when legally and practically permissible [26,134]. Such policies should prioritize traceability and scientifically defensible reuse over absolute openness.

Table 5 summarizes the priority questions, implementation requirements, validation evidence, and boundary conditions.

The immediate goal should be verified partial interoperability, not universal coverage. Starting with a small set of well-documented datasets allows mapping agreement, manual effort, provenance completeness, derivative reproducibility, and cross-dataset effects to be measured directly. Failed mappings and absent performance gains are valid results because they define where interoperability ends.

### 8.7. Limitations of the Review

This review has several limitations that should be considered when interpreting the synthesis. Screening, evidence-focused curation, full-text verification, extraction, and transparency scoring were conducted sequentially by a single reviewer; therefore, no independently duplicated screening process or inter-rater agreement coefficient was available. The intended workflow also anticipated further assessment of the 578 records classified as Unclear, but these records were not advanced to the evidence-focused second stage. This deviation was not an eligibility determination and may have introduced selection bias. In addition, the second-stage curation used judgments about redundancy and incremental contribution to construct a coherent evidence corpus. The resulting 129 publications should consequently be interpreted as a curated synthesis corpus rather than as an exhaustive population of all EEG-BCI publications that might satisfy the broader eligibility criteria. Percentages and evidence-map frequencies derived from this corpus describe the retained publications only and should not be interpreted as field-wide prevalence estimates.

Further limitations arise from the scope and heterogeneity of the evidence. The review was restricted to English-language peer-reviewed publications within the defined 2014–2026 period, apart from explicitly identified contextual sources, and it combined dataset papers, empirical studies, benchmarks, software or standards contributions, and reviews with different reporting expectations. Some conceptual themes may therefore be represented by both primary studies and review-level syntheses. The ten-item transparency score was intentionally unweighted and is publication-type dependent; it measures breadth of reporting rather than methodological quality, risk of bias, certainty of evidence, or open-science completeness. A conventional risk-of-bias synthesis and meta-analysis were not undertaken because the review questions and outcomes were heterogeneous. Finally, publication-level evidence mapping and fragmentation coding required interpretive classification even though predefined categories, evidence notes, conservative handling of missing information, and an auditable extraction trail were used. These limitations constrain claims about prevalence and completeness, but they do not negate the recurring structural, semantic, procedural, human/contextual, and computational fragmentation mechanisms documented across the retained evidence.

## 9. Conclusions

The synthesis of the curated 129-publication corpus indicates that public EEG-BCI datasets cannot be treated as a uniform, monolithic collection simply because they are openly accessible or share a nominal paradigm label. Datasets generated across diverse laboratories and hardware platforms exhibit profound multidimensional fragmentation, spanning structural, semantic, procedural, computational, and human-contextual levels [1,2,3,4,5,6,7,8,9,10,11,12,13]. These variations extend far beyond basic sensor configurations and file formats, fundamentally altering event design, experimental timing, and preprocessing pipelines. Complex user states, such as mental fatigue, motivation, overall health, and specific recording environments, are integral to how these physiological signals are generated. Consequently, they must be explicitly accounted for whenever datasets are compared or combined [38,39,40,41,42,43,44,45,46,47,48,49,50,51,52,53,54,55,56,57,58,59,60,61,62,63,64,65,66,67,68].

The feasibility of robust cross-dataset analysis remains severely constrained by incomplete data provenance, inconsistent validation strategies, and a systemic lack of executable computational resources [14,15,31,32,33,34,35,36,37]. Within the retained corpus, the median transparency score was 9/10; data availability was stated in 61.2% of publications, while code or analysis pipeline availability was stated in 20.9%. While existing structural foundations, such as EEG-BIDS, shared ontologies, and standardized benchmarking platforms are vital for organizing heterogeneous data [18,19,26,27,29,69,70,71,72,73,131,132,133,134], they each address isolated layers of the broader interoperability challenge. Similarly, although advanced statistical alignment and transfer-learning techniques successfully minimize distributional shifts across subjects and datasets, they cannot guarantee that the underlying behavioral tasks, contextual states, or class labels are genuinely semantically equivalent [74,109,110,111,112,113,114,115,116].

Overall, the aggregated evidence points to a critical need for a unified analytical framework capable of harmonizing diverse EEG-BCI resources without resorting to a forced, universal conversion process that erases meaningful dataset heterogeneity. To be effective, this framework must interlink core signal structures and experimental semantics with rich participant context and machine-actionable preprocessing provenance. For any given analysis, it must systematically evaluate which dataset components are immediately compatible, which necessitate rigorously documented transformations, and which are fundamentally unsuitable for integration. Achieving scalable EEG-BCI machine learning will require more than just algorithmic innovation; it will depend on pairing these advanced models with semantically interpretable datasets, auditable transformations, explicit uncertainty tracking, and strict evaluation protocols that preserve individual dataset identities while clearly delineating the boundaries of cross-dataset reuse.

## Figures and Tables

**Table 1:** Representative public EEG/BCI datasets and characteristics relevant to cross-dataset reuse.

Study/Dataset	Paradigm/Context	Participants and Sessions	EEG Channels and Sampling	Principal Task Design	Data Organization and Fragmentation-Relevant Feature
Wang et al. [1]	SSVEP benchmark	35 participants; one experiment, six blocks	64 channels; 250 Hz	Forty frequency- and phase-coded visual targets	Public benchmark. The frequency grid, phase coding, and display protocol are integral to dataset meaning.
Cho et al. [2]	Motor imagery	52 participants; one principal recording session	64 channels; 512 Hz	Left- and right-hand MI, real movement, resting and artifact recordings	GigaDB release with questionnaire, EMG, and three-dimensional electrode-location information; metadata-rich but principally single-session.
Kaya et al. [3]	MI and related BCI interaction states	13 participants; 75 sessions and 201 recording segments	19 EEG leads; mainly 200 Hz, with some 1000 Hz recordings	Several imagery paradigms including limb, tongue, and finger tasks	MATLAB/Figshare resource with custom synchronization markers. Sampling and recording structure vary within the resource.
OpenBMI [4]	MI, ERP/P300, and SSVEP	54 participants; two sessions on separate days	62 channels; 1000 Hz	Three BCI paradigms with offline and online phases	GigaDB data and OpenBMI toolbox; includes resting, artifact, EMG, and questionnaire data. The multi-paradigm protocol increases semantic complexity.
Zhang et al. [5]	RSVP target detection	64 participants; four blocks	64 channels; 1000 Hz acquired, 250 Hz distributed	Rare target images presented in 10 Hz visual streams	MATLAB continuous data with triggers and electrode positions. Acquisition and distributed sampling rates differ.
Ma et al. [7]	Cross-session motor imagery	25 participants; five sessions on five days	32 released channels; 250 Hz	Left- and right-hand MI, 100 trials per session	BIDS-organized EDF plus MATLAB trial files. Raw and analysis-oriented representations must be distinguished.
Won et al. [8]	RSVP and P300 speller	55 participants; one experimental visit	32 channels; 512 Hz	Resting state, RSVP target detection, and row-column P300 spelling	Figshare/GitHub release. The same participants performed two ERP paradigms, but event mappings and classification units remain task-specific.
Dreyer et al. [9]	Motor imagery with user profiles	87 participants; one session	27 channels; 512 Hz	Left- and right-hand MI with calibration and feedback phases	Zenodo/OpenViBE resource with demographic, personality, cognitive, experiential, and quality information; unusually rich participant context.
COG-BCI [10]	Passive BCI and cognitive tasks	29 participants; three sessions	64 active electrodes; 500 Hz	MATB-II, N-back, psychomotor-vigilance, and Flanker tasks	BIDS-organized EEGLAB SET/FDT files with behavior, questionnaires, and exact sensor positions. Task-specific labels require contextual interpretation.
MMV [11]	Multimodal vigilance during RSVP and SSVEP	18 participants; four sessions	62 EEG analysis channels; 1000 Hz acquired	RSVP target retrieval and prolonged SSVEP cursor control	CNT, EDF, and MAT files with raw, preprocessed, trial, and feature layers. Modalities and reference choices differ by processing stage.
Yang et al. [12]	Multi-day motor imagery	62 participants in total; three sessions	58 processed channels; 250 Hz	Two-class hand-grasp MI and a three-class variant including foot imagery	EEG-BIDS metadata with raw BDF and processed MATLAB files. Class structure differs across participant subsets.
Gu et al. [124]	Wide-frequency SSVEP	30 participants; four sessions on different days	64 channels; 1000 Hz acquired, 250 Hz epochs	Visual stimulation from 1 to 60 Hz at two modulation depths	EEG-BIDS/EDF with MATLAB and CSV derivatives plus comfort and preference ratings; broad stimulus space and user-experience metadata.

Abbreviations: BIDS, Brain Imaging Data Structure; ERP, event-related potential; MI, motor imagery; RSVP, rapid serial visual presentation; SSVEP, steady-state visual evoked potential. Values refer to the public representation described in the source publication; acquisition and distributed sampling rates may differ.

**Table 2:** Cognitive and user-related variability factors in EEG-BCI research and their implications for reproducibility and interoperability.

Factor	Typical Representation or Measurement	Role in BCI Research	Evidence Represented in the Review Corpus	Reproducibility and Interoperability Implication
Inter-subject variability	Between-participant differences in EEG features, classification performance, calibration demand, strategy, and participant descriptors	Source of performance dispersion; target of subject-independent learning and transfer	Sensorimotor variability reviews and empirical comparisons; demographic, cognitive, and experiential profiling [4,9,38,39,40,67,68]	Participant covariates and their definitions must remain linked to recordings; statistical alignment does not establish equivalence of strategy or state.
Session-to-session variability	Repeated recordings across days or sessions; session identifiers; cross-day validation; calibration records	Temporal drift, learning, habituation, and recalibration problem	Multi-day MI and passive BCI datasets; cross-day workload and vigilance studies [7,10,12,39,60,62,121]	Session order, interval, electrode setup, training, and state metadata are needed to distinguish physiological from procedural drift.
Mental fatigue	Self-report, time-on-task, behavioral performance, spectral indices, ERP changes, or derived fatigue labels	Potential confound in active/reactive BCI; target variable in fatigue-monitoring systems	P300, SSVEP, MI, pediatric, and sustained-attention studies [41,42,43,44,45,46,47,48,49,50,51]	Labels are not interchangeable unless induction, measurement, timing, and aggregation rules are documented.
Cognitive load and workload	Task difficulty or condition, subjective ratings, performance measures, physiological indices, and discrete or continuous labels	Primary target in passive BCI; possible unmeasured state in active tasks	Laboratory, air-traffic-control, driver, cross-task, and cross-day studies [52,53,54,55,56,57,58,59,60,61,62,63,64,126]	Construct names can conceal task-specific semantics; transfer requires explicit description of induction and validation procedures.
Attention and vigilance	Task blocks, response lapses, reaction-time criteria, sustained-attention measures, or continuous estimates	Target of passive monitoring; determinant of response stability in reactive BCI	Multi-task cognitive datasets, multimodal vigilance, long-term attention, and cross-session estimation [10,11,51,62]	Temporal resolution, epoching, and label-construction rules must be preserved; otherwise nominally identical vigilance classes may differ.
Motivation, emotion, and mental state	Questionnaires, self-report, experimental condition, or post hoc association with performance	Influence on engagement, strategy, and control outcome	User-state and sensorimotor-rhythm studies [65,66,67]	Frequent absence from metadata limits attribution of low performance and can confound comparisons between cohorts.
Age, health status, and cognitive function	Demographic or clinical grouping, cognitive assessments, pediatric/adult cohorts, and health-state transfer	Boundary condition for generalization and ecological validity	Pediatric fatigue and benchmarking, cross-health-state transfer, and systematic synthesis [49,68,103,125]	Cohort definitions and assessment procedures must be explicit; demographic variables should not be treated as deterministic proxies for BCI ability.
Training, experience, and ecological context	Practice history, feedback, strategy instruction, online/offline phase, laboratory/field setting, and wearable acquisition	Shapes task learning and determines the conditions under which a system is expected to operate	Training standardization, user-profile datasets, dry/wearable recordings, and realistic operational tasks [9,13,53,54,64,115,126,130]	Training and use context are part of dataset meaning; removing them during harmonization can reduce external validity.

The categories are not mutually exclusive. A factor may be the target of a passive BCI, a source of within- or between-participant variation, or both. ERP, event-related potential; MI, motor imagery; SSVEP, steady-state visual evoked potential.

**Table 3:** Sources of reproducibility failure in EEG-based BCI and minimum reporting or control requirements.

Reproducibility Layer	Recurrent Failure Source	Consequence	Minimum Reporting or Control Requirement
Data identification and access	Dataset name or version unclear; links, licenses, or persistent identifiers absent	The analyzed data cannot be located reliably, or later versions cannot be distinguished	Dataset title, repository, persistent identifier, version, access date, license, and any local exclusions
Acquisition and sensor representation	Incomplete channel, reference, coordinate, amplifier, unit, or trigger information	Signal differences cannot be separated from hardware and montage effects	Device and software versions, sampling rate, units, reference, channel list, coordinates, trigger method, and acquisition changes
Preprocessing and provenance	Filter, artifact, epoching, normalization, or operation order incompletely described	The numerical input to the model cannot be reconstructed; data leakage may go undetected	Ordered pipeline with parameters; fitting scope for every data-dependent operation; handling of bad channels, trials, and participants
Task and event semantics	Paradigm labels used without precise task, stimulus, timing, or class definitions	Nominally similar datasets may encode different neural or behavioral events	Instructions, stimuli, event dictionary, trial boundaries, timing, target definitions, feedback, and label-generation procedure
Participant and session context	Training, fatigue, workload, health status, session order, or interval not recorded consistently	Human and temporal effects are confounded with acquisition or algorithmic differences	Participant criteria, relevant covariates, session schedule, training history, calibration, and state-measurement instruments
Partitioning and validation	Trial-level leakage, unclear unit of splitting, non-nested tuning, or preprocessing fitted before splitting	Performance is optimistic or does not correspond to the claimed deployment setting	Subject/session/trial partition rules, fold construction, nesting, repeats, seeds, and strict separation of training, validation, and test data
Code and computational environment	Code unavailable or detached from the executed versions, dependencies, and parameters	Independent reruns yield different outputs or cannot be completed	Archived code revision, configuration files, software and package versions, environment or container, hardware assumptions, and random seeds
Metrics and benchmark configuration	Inconsistent metrics, aggregation, exclusions, baselines, or offline/online assumptions	Reported values appear comparable although they answer different questions	Metric definitions, class balance, dataset-level results, uncertainty, statistical tests, benchmark version, baselines, and evaluation mode

**Table 4:** Existing interoperability and standardization approaches in EEG-based BCI and their remaining limitations.

Approach or Layer	Representative Resources	Primary Contribution	Strength for Reuse	Remaining Limitation for Semantic Interoperability
Real-time stream synchronization	LSL [131]	Time-stamped transport and synchronization of heterogeneous streams	Supports multimodal acquisition and common temporal reference	Does not define the meaning of signals, markers, tasks, or user states; device latency outside the software layer may remain
Experimental BCI platforms	BCI2000 [132]; OpenViBE [133]; MetaBCI [71]; BrainFusion [72]	Reusable modules for acquisition, processing, stimuli, feedback, and deployment	Reduces implementation variation within a platform and preserves executable configurations	Representations and exports remain platform-specific; configuration consistency does not ensure cross-dataset semantic equivalence
Dataset organization and metadata	EEG-BIDS [26]	Common dataset structure and standardized descriptive fields	Improves findability, validation, automated loading, and structural consistency	Task, event, calibration, cognitive-state, and processing semantics may remain incomplete or dataset-specific
Executable processing provenance	Containerized EEG workflows [69]; BrainFusion [72]	Preservation of software dependencies, pipeline steps, and deployment configuration	Improves repeatability of encoded processing and reduces environment drift	Cannot recover undocumented acquisition conditions or missing semantic metadata
Common models and ontologies	BCI-O [70]; IEEE P2731 functional model [27]; common system/data description [29]	Formal representation of BCI entities, components, context, and relationships	Can separate concepts from local labels and support explicit semantic mappings	Coverage, adoption, legacy-data mapping, conformance testing, and representation of uncertainty remain limited
Benchmarking frameworks and dataset interfaces	MOABB [18,19]; SpeechBrain-MOABB [14]	Common loaders, paradigms, pipelines, and evaluation procedures across public datasets	Reduces duplicated implementation and enables multi-dataset comparison	Adapters may encode unexamined assumptions; a common interface can conceal differences in events, trials, and participant structure
Structural harmonization	Dimensionality transcending [28]; reference interpolation [73]; normalization [32]	Alignment of channel spaces, references, dimensions, or numerical scales	Makes heterogeneous inputs computationally compatible for joint analysis	Transformations may lose information and do not establish equivalence of tasks, labels, or participant context
Statistical transfer and domain adaptation	Cross-subject/session/dataset methods [21,22,23,24,25,75,76,77,78,79,80,81,82,83,84,85,86,87,88,89,90,91,92,93,94,95,96,97,98,99,100,101,102,103,104,105,106,107,108,109,110,111,112,113]	Reduction in distribution shift and calibration requirements	Can reuse information across users, sessions, devices, or datasets	Statistical alignment is not semantic alignment; success and failure are difficult to interpret when dataset meaning is underspecified

**Table 5:** Priority research directions for semantic interoperability and scalable EEG-BCI learning.

Research Priority	Core Question	Minimum Implementation Requirement	Evidence Needed for Validation	Principal Risk or Boundary Condition
Formal compatibility model	When are two dataset components equivalent, transformable, or incompatible for a specified analysis?	Explicit entities, relations, mapping rules, uncertainty, and information-loss representation	Expert agreement; conformance tests; known positive and negative mapping cases; analysis-specific metrics	A universal schema may conceal ambiguity or force scientifically invalid equivalence
Auditable harmonization	Can heterogeneous data be transformed while preserving source identity and scientific provenance?	Versioned transformation graph linking native data, parameters, rejected observations, and derivatives	Re-execution across environments; comparison with expert workflows; error and effort measurements	Automated conversion may propagate incorrect assumptions or obscure irreversible information loss
Cognitive/context representation	Which participant and session variables are necessary to interpret or transfer EEG-BCI data?	Minimum context profile with timing, instruments, missingness, and trait/state distinction	Repeated-session and multi-site studies; metadata ablation; subgroup analysis	Excessive metadata burden, inconsistent instruments, and privacy or access constraints
Compatibility-aware learning	Does semantic source selection improve transfer beyond statistical alignment alone?	Source filtering or weighting linked to explicit compatibility rules and dataset provenance	External-dataset tests; mismatched controls; metadata ablation; positive and negative transfer analysis	Metadata may be incomplete, correlated with site, or insufficient to predict transferability
Cross-dataset benchmarks	Do models generalize to independently collected datasets without leakage or hidden overlap?	Versioned dataset registry, safe partitions, leave-one-dataset-out evaluation, per-dataset reporting	Reproducible pipelines; external validation; uncertainty estimates; duplicate and overlap audits	Benchmark results may be dominated by dataset choice, class mismatch, or unreported preprocessing
Governance and adoption	Can the representation be maintained, extended, and used across tools and institutions?	Validation tools, persistent identifiers, versioning, extension policy, licenses, and legacy mappings	Independent implementations; community pilots; interoperability tests across repositories and platforms	Fragmented adoption, incompatible extensions, and insufficient incentives for detailed reporting

## Data Availability

The implemented review protocol and methodological record are publicly available on OSF (https://doi.org/10.17605/OSF.IO/VS5FW). Licensed database exports are not redistributed.
